# Variation in host home range size decreases rabies vaccination effectiveness by increasing the spatial spread of rabies virus

**DOI:** 10.1111/1365-2656.13176

**Published:** 2020-02-15

**Authors:** Katherine M. McClure, Amy T. Gilbert, Richard B. Chipman, Erin E. Rees, Kim M. Pepin

**Affiliations:** ^1^ United States Department of Agriculture, Animal and Plant Health Inspection Service National Wildlife Research Center Fort Collins CO USA; ^2^ Department of Microbiology, Immunology, and Pathology Colorado State University Fort Collins CO USA; ^3^ United States Department of Agriculture, Animal and Plant Health Inspection Service National Rabies Management Program Concord NH USA; ^4^ Land and Sea Systems Analysis Inc. Granby QC Canada; ^5^ National Microbiology Laboratory Public Health Risk Sciences Division Public Health Agency of Canada Saint‐Hyacinthe QC Canada; ^6^ Present address: Cornell Atkinson Center for Sustainability and the Cornell Wildlife Health Center Cornell University Ithaca NY USA

**Keywords:** home range, ORV, rabies, raccoon ecology, spatially explicit model, vaccination

## Abstract

1. Animal movement influences the spatial spread of directly transmitted wildlife disease through host–host contact structure. Wildlife disease hosts vary in home range‐associated foraging and social behaviours, which may increase the spread and intensity of disease outbreaks. The consequences of variation in host home range movement and space use on wildlife disease dynamics are poorly understood, but could help to predict disease spread and determine more effective disease management strategies.
2. We developed a spatially explicit individual‐based model to examine the effect of spatiotemporal variation in host home range size on the spatial spread rate, persistence and incidence of rabies virus (RABV) in raccoons (*Procyon lotor*). We tested the hypothesis that variation in home range size increases RABV spread and decreases vaccination effectiveness in host populations following pathogen invasion into a vaccination zone.
3. We simulated raccoon demography and RABV dynamics across a range of magnitudes and variances in weekly home range size for raccoons. We examined how variable home range size influenced the relative effectiveness of three components of oral rabies vaccination (ORV) programmes targeting raccoons—timing and frequency of bait delivery, width of the ORV zone and proportion of hosts immunized.
4. Variability in weekly home range size increased RABV spread rates by 1.2‐fold to 5.2‐fold compared to simulations that assumed a fixed home range size. More variable host home range sizes decreased relative vaccination effectiveness by 71% compared to less variable host home range sizes under conventional vaccination conditions. We found that vaccination timing was more influential for vaccination effectiveness than vaccination frequency or vaccination zone width.
5. Our results suggest that variation in wildlife home range movement behaviour increases the spatial spread and incidence of RABV. Our vaccination results underscore the importance of prioritizing individual‐level space use and movement data collection to understand wildlife disease dynamics and plan their effective control and elimination.

## Graphical abstract

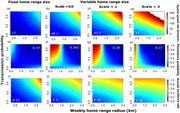

The authors developed a spatially explicit model of raccoon population and rabies virus infection dynamics, and show that variation in host home range size increases rabies spread and incidence, decreasing the population‐level effectiveness of oral rabies vaccination. The results underscore the importance of host movement and space use heterogeneity on disease dynamics and elimination efforts.

## 1 INTRODUCTION

Animal movement is a key component of many ecological processes, including population dynamics, species interactions and the spatial spread of infectious wildlife diseases (Bowler & Benton, 2005; Hess, 1996; Kays, Crofoot, Jetz, & Wikelski, 2015). Natural and human‐mediated movements of infected domestic animals and wildlife have been implicated in the spread of diseases such as bovine tuberculosis (TB) in cattle and possums, chronic wasting disease in mule deer and rabies in raccoons (Corner, Stevenson, & Collins, 2003; Farnsworth, Hoeting, Hobbs, & Miller, 2006; Gilbert et al., 2005; Rosatte et al., 2006). For directly transmitted pathogens, host movement influences the spatiotemporal distribution of host**–**host contact, and underpins the contact structure between infectious and susceptible individuals (Morales et al., 2010). Animal movement can play critical direct and indirect roles in pathogen transmission, yet our understanding of how spatiotemporal or individual‐level differences in natural wildlife host movement affects disease dynamics is limited.

Effects of variation in wildlife movement on the transmission of directly transmitted wildlife pathogens depend on the interplay of host ecology, pathogen ecology and the spatial structure of host contact. Host variability in contact rates, susceptibility, infectiousness or spatiotemporal variability in other host characteristics related to pathogen transmission can increase both the intensity of disease outbreaks and probability of pathogen extinction (Lloyd‐Smith, Schreiber, Kopp, & Getz, 2005; Woolhouse et al., 1997), and are common in both human and wildlife populations (Paull et al., 2012; VanderWaal & Ezenwa, 2016). Contact heterogeneities in wildlife populations can arise from complex social structure or fluctuations in the spatial distribution of hosts (Craft, 2015; Drewe, 2010; Hamede, Bashford, McCallum, & Jones, 2009). Simulations of personality‐dependent individual‐level movement variation in animals suggest movement variation influences animal contact rates (Spiegel, Leu, Bull, & Sih, 2017), and consistent individual‐level variation in wildlife movement related to natal dispersal and foraging tactics have been documented (Bonnot et al., 2015; Clobert, Baguette, Benton, & Bullock, 2012). If spatiotemporal variation in host movement promotes heterogeneity in the capacity for individuals to contact or transmit pathogens to other hosts, host movement variation could result in transmission heterogeneity that increases wildlife disease spread and incidence while decreasing pathogen persistence. Understanding the effects of host movement variation on wildlife disease dynamics could thus be critical for predicting spatial spread (Cross et al., 2010).

Host movement and space use also influence the effectiveness of wildlife disease intervention strategies. For example, the explicit consideration of red fox (*Vulpes vulpes*) territoriality and resulting patterns of conspecific density was crucial for the elimination of red fox rabies in Western Europe, underscoring the importance of host home range movement when targeting free‐ranging wildlife species for disease elimination (Freuling et al., 2013; Murray et al., 1986). Conversely, disease management strategies can affect animal movement and lead to unintended consequences for pathogen transmission. For instance, badger culling to reduce spillover of bovine TB to cattle in the United Kingdom increased badger dispersal movement, contact rates and transmission to cattle near the culling zone (Donnelly et al., 2006; Pope et al., 2007). Ultimately, targeted control measures that treat or eliminate individuals that are most connected could be more effective than applying interventions randomly (Lloyd‐Smith et al., 2005). In this context, an understanding of how variation in and scope of host home range movement and space use influence wildlife disease management strategies can be important for planning effective vaccination efforts.

Rabies virus (RABV) is a zoonosis caused by single‐stranded RNA viruses of the genus *Lyssavirus* (Wunner, 2007). Transmission occurs primarily through bite contact among hosts, and infectious mammals invariably develop fatal encephalomyelitis (Rupprecht, Hanlon, & Hemachudha, 2002). Raccoon RABV is the most prevalent variant of RABV in the United States, with raccoons (*Procyon lotor*) accounting for the highest proportion of rabid wildlife during 1991**–**2014 (Ma, 2018). The objectives of the US raccoon rabies management program are to prevent the westward expansion of and eliminate this specific RABV variant, primarily by deploying oral vaccine baits that provide long‐term immunity to raccoons against RABV infection when ingested (Blanton et al., 2018; Slate et al., 2009). Oral rabies vaccination (ORV) has proven effective for the elimination of canine RABV in coyotes in the United States, raccoon RABV in Canada and red fox RABV throughout Western and Central Europe (Müller et al., 2015; Rosatte et al., 2009; Sidwa et al., 2005).

We developed a spatially explicit individual‐based model (IBM) of raccoon population dynamics and RABV transmission to investigate how spatiotemporal variation in wildlife host home range movement, implemented as home range size variation, affects the spatial spread, persistence, and incidence of wildlife disease and vaccination effectiveness. We hypothesized that variable home range size would increase pathogen spread and incidence rates, and decrease pathogen persistence, compared to conditions assuming fixed home range size, as predicted by theory (Lloyd‐Smith et al., 2005). We tested the hypothesis that variation in host home range size decreases vaccination effectiveness in wildlife host populations following the invasion of RABV into an ORV zone, and examined the relative effectiveness of ORV strategies targeting raccoons. We predicted that fall vaccination would be more effective than spring vaccination because young of the year would be old enough to consume ORV baits in the fall but not late spring (Wandeler, 1991).

## 2 MATERIALS AND METHODS

### 2.1 Model design

#### 2.1.1 Approach

We modelled raccoon demographics and RABV infection dynamics using a spatially explicit, discrete‐time IBM to examine the role of variable home range size in the spatial spread of RABV and ORV effectiveness. We compared effects of variable versus fixed host home range sizes across a range of magnitudes to identify when home range size variation—implemented as week‐to‐week stochastic changes in host home range radii in km—had the strongest effects on spatial RABV spread rates and ORV effectiveness. We modelled home range size variation in the context of additional complexities of raccoon ecology, including demography and social structure, to account for their effects on disease processes and intervention outcomes. We describe key components of the model below and provide additional details using the updated Overview, Design Concepts, and Details protocol for IBMs (Grimm et al., 2010) in the Supporting Information.

#### 2.1.2 Spatial design

**Figure 1:**
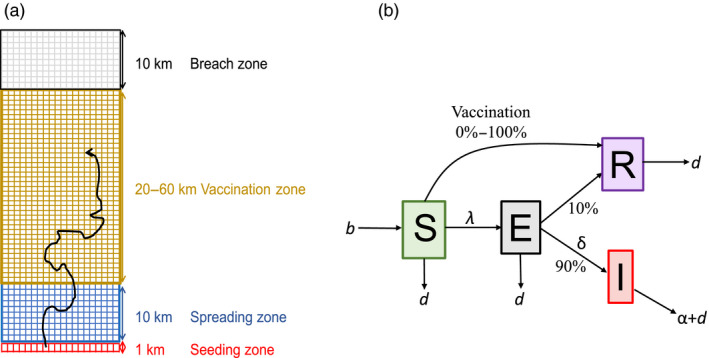
Landscape and disease components of model. (a) Landscapes consisted of 1 km^2^ grid cells with four zones. Raccoons infected with rabies virus were introduced to the seeding zone; breach occurred when an infectious individual crossed into the breach zone. (b) Transitions between S (susceptible), E (exposed), I (infectious) and R (recovered) disease states are governed by the force of infection (λ), incubation rate (*δ*) and disease‐induced mortality rate (*α*). Demographic rates include birth (*b*) and natural death (*d*)

Simulated landscapes contained four spatially consecutive zones composed of 1 km^2^ gridded cells: seeding (1 × 20 km), spreading (10 × 20 km), vaccination (20**–**60 × 20 km) and breach (10 × 20 km), for a total landscape area ranging between 820 and 1,620 km^2^ (Figure 1a). Individuals were assigned a randomly drawn home range centroid point located in continuous space within a discrete grid cell. Cell‐level carrying capacity was 15 individuals/km^2^, corresponding to typical suburban raccoon densities (Table S1). We tracked disease‐related and demographic characteristics of each individual on a weekly time‐step. Simulations were conducted in Matlab R2016b (Version 9.1.0, MathWorks, Inc.). Results were analysed in R v3.4.2 (R Core, 2017).

#### 2.1.3 Demography, natal dispersal and social structure

We modelled reproduction as a single 6‐week birth pulse from April to mid‐May (see Table S1 and Supporting Information Methods for more details). Individuals were subject to density‐dependent mortality to maintain densities at or below within‐cell carrying capacity. If cell‐level carrying capacity was exceeded, individuals within the cell were randomly chosen and removed from the simulation, with younger individuals taken first to mimic observed patterns in juvenile and adult survivorship (Gehrt & Fritzell, 1999). We modelled male‐biased natal dispersal as two random variables, natal dispersal distance and dispersal age (described in Supporting Information). Individuals that moved off the landscape during dispersal were lost permanently, and did not move back onto the landscape. We modelled social structure comprising family groups of females and offspring, male dyads, and solitary males. Field and genetic studies suggest that daughters may associate with mothers and her offspring into adulthood (Cullingham et al., 2008; Gehrt & Fritzell, 1998), while males often associate in relatively long‐lasting, non‐familial dyads followed by separation to become independent as they mature (Gehrt & Fritzell, 1998; Gehrt, Gergits, & Fritzell, 2008). We assumed that individuals in the same family group shared the same home range centroids and had higher transmission probabilities within a family group relative to between family groups (Table S1).

#### 2.1.4 Weekly contact

We modelled variation in home range size as a random variable described by a gamma distribution of weekly varying home range radii (in km). We used parameters derived from (or similar to) maximum weekly distances moved by raccoons, which were estimated from GPS relocation data obtained from 26 free‐ranging raccoons captured in a suburban ORV area of Burlington, VT (Table S1; United States Department of Agriculture Animal and Plant Inspection Service [USDA APHIS], Wildlife Services, unpublished data). GPS locations, or fixes, were recorded every 30 min to 2 hr from 6 p.m. to 6 a.m. from late July through mid‐September 2016. For each individual, we calculated the maximum distance (in km) between all fixes within a week, which we considered to be a measure of an individuals’ maximum weekly exploratory potential. We modelled weekly home range size variation because we observed individual‐level variation in maximum distances moved at this temporal scale, and because it was relevant for RABV transmission. We fit a gamma distribution to observed distances using maximum likelihood methods (mean = 0.82 km, median = 0.75 km, variance = 0.16). We used a second, theoretical gamma distribution with a higher variance to explore how more variable home range size affected vaccination effectiveness (mean = 1 km, median = 0.84 km, variance = 0.5). A home range radius was randomly assigned to each susceptible individual relative to their fixed home range centroid at each time step to delineate a home range area within which host**–**host contact occurred (Figure S1). We assumed individuals explored the entirety of their weekly home ranges, and that home range movement scaled proportionally with home range size. For scenarios where animals had a fixed home range size, all individuals were assigned the same home range radius throughout the simulation. Contact opportunities in the home range were assumed equally probable given the high degree of social connectivity observed in suburban raccoon populations (Hirsch, Prange, Hauver, & Gehrt, 2013).

#### 2.1.5 Disease transmission

We modelled rabies disease dynamics with four disease states: susceptible (S), exposed but not infectious (E), infectious (I) and recovered (R; Figure 1b). Density‐dependent transmission occurred when home range centroids of infectious individuals were within the weekly home range size of a susceptible individual, according to a fixed transmission probability given contact. Transmission probability was based on family group membership to reflect potential differences in within‐ versus between‐group contact rates in raccoons (Figure S1). Within‐group transmission probability was fixed at 0.5, whereas between‐family transmission probability ranged between 0.001 and 0.5. Within‐group transmission was non‐spatial because we assumed weekly contact probability was 100% within family groups, whereas between‐group transmission was spatially explicit because contact required that home range centroids of infectious individuals were in the weekly home range of a susceptible individual. Recovery rate of exposed individuals was 10% to capture variation in levels of acquired rabies immunity observed in raccoons (Slate et al., 2014). Disease‐induced mortality was 100% for infectious individuals (Hanlon, Niezgoda, & Rupprecht, 2007), and occurred 1 week after individuals transitioned from the exposed to infectious disease class (Hanlon et al., 2007). The RABV incubation period among exposed individuals was drawn from a Poisson distribution (mean = 4 weeks; Table S1).

The force of infection, or the per capita rate at which susceptible individuals seroconvert to the exposed class, $\lambda_{t}$ at week *t*, was:

$$\lambda_{t}=\sum_{i\;=\;1}^{N}\sum_{w\;=\;1}^{K}S_{i}I_{w}\beta_{\text{within}}+\sum_{i\;=\;1}^{N}\sum_{b\;=\;1}^{K}S_{i}I_{b}\beta_{\text{between}}$$

where $\beta_{\text{within}}$ is within‐group transmission probability, $\beta_{\text{between}}$ is between‐group transmission probability, *w* represents individuals in the same family group as focal individual *S_i_* in week *t* and *b* represents individuals that are not in the same family group as *S_i_* in week *t* but are located within the weekly home range of *S_i_.*

#### 2.1.6 Vaccination

To model vaccination, we randomly selected a fixed proportion of animals within the vaccination zone irrespective of disease or vaccination status and transitioned susceptible and exposed animals to the recovered class with a 2‐week lag for development of vaccine‐induced immunity. We neglected factors that influence achieved vaccination coverage (e.g. baiting density, non‐target interspecific competition for baits), and assumed that any coverage could be achieved, because we were interested in exploring how variation in home range size affects ORV zone breach probabilities over a range of theoretical coverage levels. We assumed that vaccinated individuals acquired lifetime immunity with no waning. Individuals younger than 17 weeks were not vaccinated because delivery was by ORV and raccoons younger than this age may still be dependent on the dam for nourishment (Montgomery, 1969). Vaccination was assumed to be ineffective on infectious individuals.

### 2.2 Simulations

Sensitivity analyses included a full factorial design of three parameters: (a) shape, (b) scale parameters of the weekly home range radius gamma distribution and (c) between‐group transmission probability. We used four scale parameters of the gamma distribution (0.2, 0.5, 1 and 2) corresponding to increasing variance, with associated shape parameters that correspond to medians of the gamma distribution, ranging from 0.2 to 3 km in 0.2 increments (Figure S3). Between‐group transmission probabilities were evaluated from 0.001 to 0.5 in logarithmic intervals for a total of 600 parameter sets. For static home range size simulations, we examined effects of a fixed home range radius on outbreak dynamics by varying the fixed radius from 0.2 to 3 km in 0.2 increments for an additional 150 parameter sets. All simulations included a 1‐year demographic transient period followed by the exposure of all hosts located in the middle grid cell of the seeding zone to RABV (~15 individuals, early spring, week 11). Simulations occurred on a 1,220 km^2^ landscape without vaccination. We ran 100 eight‐year simulations per parameter set for a total of 75,000 simulations.

**Figure 2:**
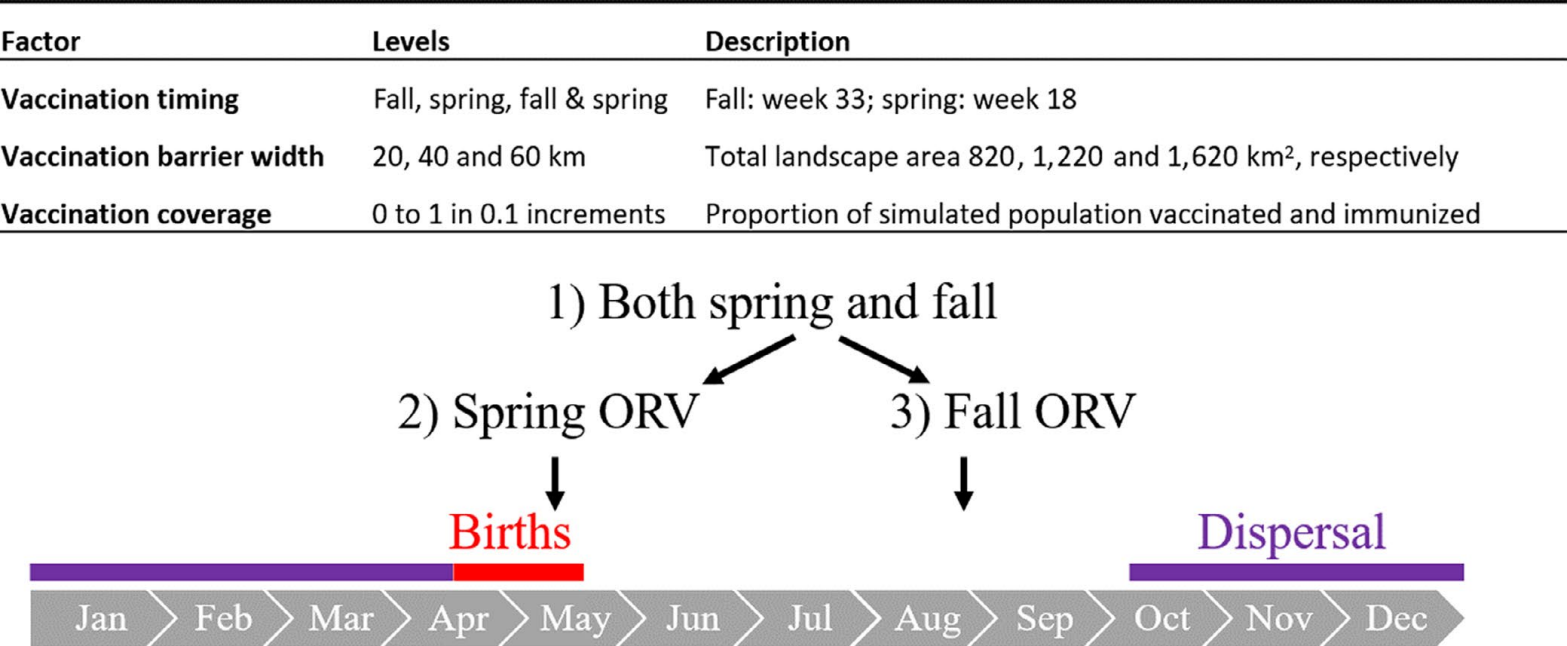
Components of vaccination. The timing and frequency of oral rabies vaccination (ORV) in relation to the annual birth pulse and male‐biased natal dispersal is shown

To explore the effect of home range size variation and ORV strategies on the probability that RABV will breach a vaccination zone, we modelled vaccination in a separate set of simulations. We ran all combinations of two between‐group transmission probabilities (0.05 and 0.1), two distributions that described weekly home range radii (described above), and three components of ORV deployment: (a) vaccination coverage or the proportion of animals immunized within the ORV zone, (b) timing and frequency of vaccine application (fall, spring or both fall and spring) and (c) ORV zone width (20, 40 and 60 km, Figures 1a and 2). In the United States, 40 km is the standard ORV zone width used by managers targeting raccoons. We modelled vaccination coverage ranging from 0% to 100% in 10% increments, where 0% comprised no vaccination control (Figure 2). Vaccination coverage is a key component of intervention effectiveness as it underlies herd immunity, or the population‐level immunity required for pathogen transmission to decline (Anderson & May, 1985). We ran 396 unique parameter sets, with 100 ten‐year replicate simulations per parameter set.

### 2.3 Model outputs and statistical analysis

#### 2.3.1 Sensitivity analyses

We calculated annual spatial spread rate, pathogen persistence and per capita annual incidence as outputs of sensitivity analyses. We calculated annual spatial spread rate (km/year) as the linear distance that RABV travelled/year from the seeding zone. We restricted spread calculations to simulations where annual incidence rate was ≥0.001 because we were interested in simulations that led to ongoing transmission (i.e. avoided stochastic fade‐out at initiation). RABV persistence was calculated as a binary response where persistence was defined as the presence of at least one exposed or infectious individual in any cell at the last time step of the 8‐year simulation. Annual incidence rate was calculated as the mean annual new cases/annual maximum population size across years in which infections were present, constrained to runs where annual incidence rate was ≥0.001. We analysed outputs for fixed and variable weekly home range sizes separately using generalized linear models (GLM), with home range size variation, magnitude, transmission probability and their interactions as covariates (see Table S2 for model specifications). Specifically, covariates included median distance of the home range radius distribution (or in the fixed home range size case, the value of the constant home range radius), the scale parameter of the gamma distribution (for variable home range size simulations only) and between‐group transmission probability.

#### 2.3.2 Vaccination analyses

We defined a RABV breach of the vaccination zone as a binary response variable in which infectious individuals did or did not breach the vaccination zone during the simulation (Figure 1a). We report breach probability as the proportion of 100 simulations in which the vaccination zone was breached. We used GLMs with a binomial distribution and a logit link, with fixed effects that included vaccination timing, coverage, zone width, between‐group transmission probability and weekly home range radius distribution. We calculated vaccination effectiveness as 1 – *v*, where *v* is the minimum vaccination coverage required to reduce RABV breach probability to zero.

#### 2.3.3 Model evaluation and *R*_0_

For sensitivity and vaccination simulations, we evaluated the relative support of covariates using Akaike Information Criterion (AIC; Akaike, 1973), including all two‐way interactions, and describe relationships of responses to covariates using the best supported model for each response variable (Supporting Information). We calculated *R*_0_, the average number of transmissions from an index case in a completely susceptible population, across 1,000 two‐year replicate simulations using the data‐informed home range radius distribution and transmission probability = .05 (see Supporting Information for details).

## 3 RESULTS

### 3.1 Effects of variation in home range size on RABV spatial spread, persistence and incidence

**Figure 3:**
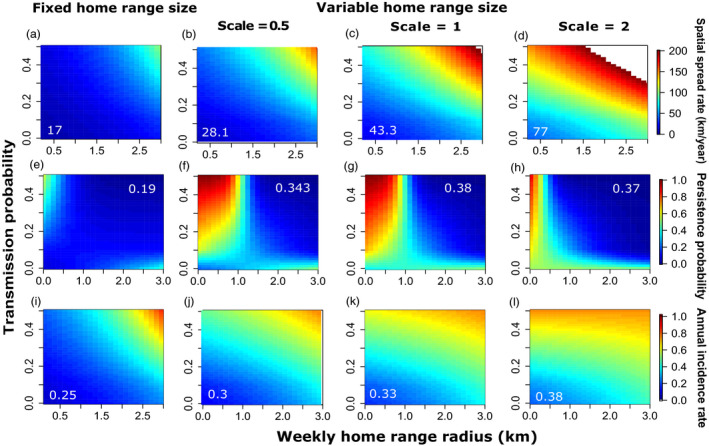
Annual spatial spread, persistence probability and annual incidence rate. Rows correspond to model outcomes with legends shown on the right. Columns correspond to levels of variation in home range size, beginning with no variation on the left followed by increasing values of the scale parameter of the host home range radius distribution, reflecting increasing variation in host home range size. Each plot shows the same range of median home range sizes along the *x*‐axis. Heat map colours are: (a–d) annual spatial spread rate (km/year), (e–h) pathogen persistence probability over the 8‐year simulations and (i–l) mean annual incidence rate (mean annual new cases/annual maximum host abundance). The mean value across all simulations is shown in white

**Figure 4:**
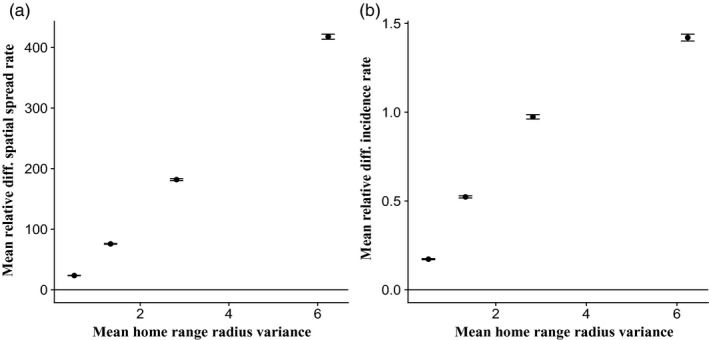
Mean relative difference of fixed versus variable home range sizes in spatial spread and incidence rates. (a) Mean relative difference in spatial spread rates (km/year) between fixed and variable home range size simulations, calculated as the average difference between variable and fixed home range results divided by fixed home range results, plotted against the mean variance of the weekly home range radius gamma distribution (for each of the 4 scale parameters). Points (±1 *SE*) are means across all medians and transmission probabilities. (b) Same as (a), but for mean relative difference in incidence rates (annual new cases/annual maximum host abundance) between fixed and variable home range results. Relative differences were evaluated where the median of the gamma distribution = fixed home range radius, in km

**Figure 5:**
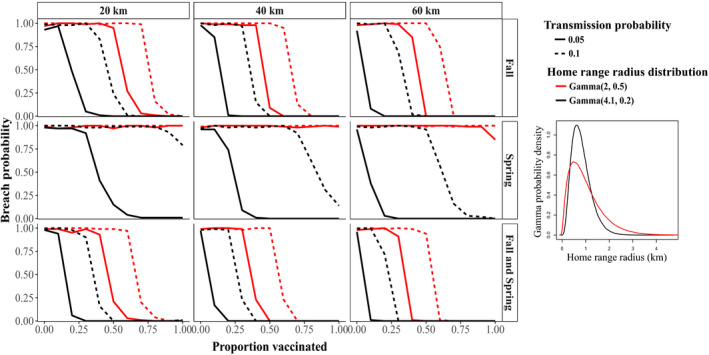
Breach probability given different vaccination strategies and variable home range sizes. Columns correspond to vaccination zone widths (20, 40 or 60 km) and rows to vaccination timing (fall, spring or fall and spring). Line colour indicates the two home range radius gamma distributions (in km) which describe the variation in weekly home range size; black is data‐informed and red is hypothetical. Line type indicates between‐group transmission probabilities. Vaccination coverage is indicated along the *x*‐axes. Insert shows gamma probability density functions (with parametrization shape and scale) implemented as weekly varying home range radii

Variation in the weekly home range size increased spatial spread rates across all magnitudes of home range sizes relative to simulations assuming fixed home range size (Figures 3a–d and 4a; Tables S2 and S3). For home range radii between 0.03 and 3 km, the less variable distributions (scale parameter of the gamma distribution = 0.5, mean variance range = 0.33–0.88, inset Figure 5b) increased spatial spread rates by 176%, while more variable distributions (scale parameters of the gamma distribution = 1 and 2, mean variance range = 1.91–4.41, Figure 3c,d) increased spatial spread rates by 282%**–**518% across all transmission probabilities. The relative effect of increased variance in host range size compared to fixed home range size was most pronounced at smaller median home range sizes (Figure S4a), suggesting variable space use may most strongly affect spread rates for groups of animals with smaller home ranges. However, this spatial spread pattern may also be influenced by the relative difference in variance in high and low variance models, which is inversely related to the median size of home ranges (Figure S5). Figure 3a–d also demonstrate the effects of transmission probability on increasing rates of spatial spread, highlighting the interactive effect of home range size and transmission probability on spatial spread rates (Table S2).

Variation in the weekly home range size influenced pathogen persistence probability, which was also strongly influenced by transmission probability (Figure 3e**–**h; Tables S2 and S4). Mean persistence probability was low across much of the parameter space explored in simulations assuming fixed home range sizes (mean persistence probability = .19, Figure 3e). For smaller home range sizes (<7 km^2^—equivalent to radius <1.5 km), variation increased persistence probability by 305%**–**383% relative to fixed home ranges of the same size. For median home range sizes of 7 km^2^ (radius = 1.5 km), intermediate levels of variation (scale parameter = 1, Figure 3g) maximized persistence probability relative to more or less variable home range sizes, increasing persistence probability by 106% compared to the less variable sizes (Figure 3f), and by 110% compared to more variable sizes (Figure 3h). For larger median values of home range size (radius of 1.5**–**3 km), variation increased persistence probability between 110% and 150% relative to fixed home ranges of the same size (i.e. radii of equal length). In summary, for smaller home range sizes, intermediate levels of variation had the highest persistence probability, while for larger home range sizes, the effect of variation on persistence weakened considerably relative to simulations with fixed home range sizes.

Annual incidence rates increased with variation in host home range sizes relative to most simulations assuming fixed sizes (Figures 3i\x96l and 4b; Tables S2 and S5). For smaller home range sizes (radii < 1.5 km), variation in weekly home range sizes increased incidence rates by 167%**–**292%, whereas at larger home range sizes (radii of 1.5**–**3 km), variation increased incidence rates by 137%–192%, when gains in fixed versus variable home range sizes began to diminish. At the highest transmission probabilities, the reverse patterns were observed (Figures S4b and S5). The relative strength of the effect of variation in home range size also depended on transmission probability, as seen in Figure 3i**–**l and Table S4. Average *R*_0_ was 0.76 (95% CI (0.697, 0.827)) across 1,000 replicate simulations, and 1.72 (95% CI (1.66, 1.77)) for those simulations (444/1,000) that did not undergo stochastic fade‐out at initiation.

### 3.2 Effects of home range size variation on vaccination effectiveness

Variation in weekly home range sizes strongly influenced ORV effectiveness (Figure 5). Across all simulations, the home range sizes that had higher median values and more variation led to consistent decreases in ORV effectiveness that were driven primarily by increasing variance rather than median size (Figure S6, Supporting Information methods and results, Tables S6 and S7). For the fall‐only ORV timing, for example, home range sizes with more variation decreased relative effectiveness by 54% compared to those with less variation (red vs. black lines, Figure 5), while for spring‐only ORV timing, higher variation decreased relative effectiveness by 100%. Breach probability increased with transmission probability. In simulations with fall and spring vaccination, for example, doubling the between‐group transmission probability decreased ORV effectiveness by 21.7%–41.8% in simulations with higher or lower variation in home range sizes, respectively, where all else was held constant.

### 3.3 Effectiveness of different components of ORV deployment

In simulations with fall ORV timing, vaccination was 40% effective (Table S5), while with spring ORV timing, vaccination was 18.8% effective, when all other conditions were held constant (Figure 5). When ORV deployment occurred in both fall and spring, it was 50% effective, suggesting diminishing returns with increased vaccination frequency. With the 40 km ORV zone, for example, the fall and spring ORV timing increased relative effectiveness by 22% compared to fall‐only ORV timing, while for the 60 km zone, increasing the frequency of ORV timing increased ORV effectiveness by 9.5%.

ORV effectiveness increased with vaccination coverage, as expected (Figure 5; Table S7). On average, the minimum coverage required to reduce the probability of breach to zero was 52.8% (range = 0.2**–**1) in simulations where complete reduction was achieved. ORV effectiveness increased with increasing vaccination zone width, but had diminishing returns on breach probability compared to effects of vaccination coverage and timing. For example, the 60 km ORV area increased relative effectiveness by only 2% compared to the 40 km zone, while the 40 km zone increased effectiveness by 67% over the 20 km zone, when all other conditions were held constant.

## 4 DISCUSSION

We found that variation in raccoon home range size had large impacts on the rate of spatial spread of RABV and the effectiveness of ORV in containing vaccination zone breaches. Our results show that variation in raccoon space use can increase the spread and incidence of RABV, likely by infrequent but substantially longer distance home range movements of ‘supermover’ individuals (Craft, 2015; White, Forester, & Craft, 2017). We show that interactions between host space use and transmission probability can strongly affect epidemiological processes and vaccination effectiveness, highlighting the need for more information about factors affecting transmission probability and habitat‐associated host movement for planning effective control programmes.

Variation in host home range size influenced disease dynamics by at least two non‐mutually exclusive mechanisms in our model. First, more variable host home ranges increased spatial spread rates because susceptible individuals were likely to contact infectious individuals over longer distances, thus accelerating the advancing spread of RABV to new disease foci. This should have particular importance for species that exhibit heterogeneous population structure and/or social groupings—including lions, jackals, in addition to raccoons—because far‐ranging individuals can link spatially or socially isolated groups (Craft, Volz, Packer, & Meyers, 2011; Loveridge & Macdonald, 2001; Russell, Real, & Smith, 2006). Second, variation in host home range size may contribute to host contact heterogeneity. Far‐ranging susceptible individuals may have more contacts and thus be more likely to become infected, increasing both spread and incidence rates, consistent with the patterns we report here. Our results suggest that variable host home range size can drive spatiotemporal variation in contact rates that ultimately affect spatial spread and incidence rates, supporting a growing consensus that variation in host behaviour—including host movement and space use—strongly influence wildlife disease dynamics (Dougherty, Seidel, Carlson, Spiegel, & Getz, 2018; Newton et al., 2019; VanderWaal & Ezenwa, 2016).

Our vaccination simulations highlight several key findings for RABV management by ORV. First, we found that increases in host home range size variation sharply decreased vaccination effectiveness by increasing spatial spread and incidence rates, leading to more frequent vaccination zone breaches at lower to moderate levels of vaccination coverage. Given that a few individuals may disproportionately influence the success or failure of ORV, efforts to better understand drivers of raccoon movement (e.g. conspecific distribution, landscape, disease status) should be a priority, for both infected and uninfected animals. Clinical behaviours of infectious raccoons range from aggressiveness towards conspecifics to paralysis and impaired mobility (Jenkins & Winkler, 1987). Widely roaming infectious individuals (Roscoe et al., 1998) could disproportionately increase disease spread, while paralytic behaviour could impede pathogen transmission and slow disease spread. The outcome of pathogen‐induced movement behaviour on rabies spread may thus depend on the balance of rabid movement behaviour among raccoon populations (Reynolds, Hirsch, Gehrt, & Craft, 2015). Additionally, in uninfected or incubating individuals, individual‐level movement behaviour and associated home range space use can shift in response to disease‐induced population declines. For instance, movement patterns and contact rates of red foxes changed as population density decreased following a sarcoptic mange epizootic, leading to increased movement and larger territories (Potts, Harris, & Giuggioli, 2013). Unlike red foxes, however, raccoons exhibit a range of social tolerances—including complex seasonally varying associated and non‐associated behaviours, and strict territoriality (Chamberlain & Leopold, 2002; Gehrt & Fritzell, 1998), which might differentially influence behavioural responses to decreased conspecific density following outbreaks.

A second implication for disease management is that bait distribution in fall appears more effective at containing RABV transmission than in spring. Our simulations show seasonal disease dynamics driven by the influx of susceptible juveniles during the synchronous birth pulse in early April to mid‐May. Spring vaccination was less effective because it coincides with this birth pulse, when susceptible juveniles are not yet weaned and are unlikely to forage for and ingest oral vaccine baits (Fry et al., 2013). Fall vaccination was more effective because susceptible juveniles—who otherwise may have been infectious or incubating the virus—were immunized prior to natal dispersal. Vaccination in both the spring and fall increased effectiveness slightly, but there may be diminishing returns given the relatively small gains in effectiveness and increased implementation costs of a biannual vaccination effort. We note that gains from spring only vaccination or spring and fall together may be greater if protective maternal antibody transmission from vaccinated adult females to young—which we did not model—is prolific in this system. Other components of behaviour that we did not model, including breeding and non‐breeding contact patterns, may exhibit seasonal variation that could also influence optimal vaccination timing (Reynolds et al., 2015). Our simulations lend support to current ORV timing, but a cost‐effectiveness analysis is needed to fully assess the added utility of implementing vaccination twice rather than once per year.

One caveat to this work is that we assumed a homogeneous landscape in our simulations. Landscape heterogeneity can influence the spatial spread of wildlife and plant diseases through scale‐dependent effects on host distribution, density and movement (Meentemeyer, Haas, & Václavík, 2012). At larger spatial scales, topographical features such as mountain ranges, rivers and lakes can influence raccoon movement and partially contain or slow rabies spread among raccoons (Cullingham, Kyle, Pond, Rees, & White, 2009; Smith, Waller, Russell, Childs, & Real, 2005). At smaller spatial scales, spatial heterogeneity resulting from differences in underlying resources influence raccoon foraging behaviours, host movement and potentially disease processes (Tardy, Massé, Pelletier, & Fortin, 2018; Tardy, Massé, Pelletier, Mainguy, & Fortin, 2014). Importantly, landscape structure can have unexpected consequences on vaccination success when landscape heterogeneity affects host population dynamics and space use. For example, very low vaccination coverage could prevent rabies epizootics that threaten Ethiopian wolves when vaccination targets host dispersal corridors (Haydon et al., 2006). In contrast, low to moderate levels of immunity in raccoons could be counterproductive in landscapes with habitat heterogeneity because RABV could be perpetuated among weakly connected refuges, leading to epizootics in neighbouring areas (Rees, Pond, Tinline, & Denise, 2013). Realistic landscape heterogeneity and mechanistic movement in evaluating disease dynamics and vaccination strategies (e.g. Tracey, Bevins, Vandewoude, & Crooks, 2014; White, Forester, & Craft, 2018) are important directions for future work. A framework that accounts for landscape‐driven movement processes would be useful for identifying spatial bait distribution strategies that could increase bait exposure and seroconversion rates, and ultimately, ORV coverage and effectiveness.

We modelled variation in home range sizes on a weekly time‐scale. Dynamic, or elastic, home ranges reflect underlying spatiotemporal differences in demography, environmental conditions or territorial behaviour (Tao, Börger, & Hastings, 2016). Raccoon home range size can shift in response to underlying resources, such as concentrated food sources in urban areas (Schuttler et al., 2015) but the time‐scale of potential home range expansion and contraction remains understudied in most areas (although we note that GPS data from raccoons in Burlington, Vermont and Chattanooga, Tennessee both suggest that home range sizes varied weekly, USDA APHIS Wildlife Services, unpublished data). Our results suggest that dynamic home range sizes resulting from fluctuating resources could increase pathogen transmission and RABV spatial spread in resource‐subsidized raccoon populations.

A final caveat to this work is that we assumed hosts explored their home range fully and homogenously. This ignores the potential for underlying habitat differences that could affect foraging behaviours, movement and contact heterogeneity. Recent advances in analytical approaches for studying wildlife space use, including mechanistic home range movement models that connect underlying movement, resource selection, territoriality and spatial utilization patterns, are advancing understanding of the behavioural underpinnings of home range animal movement (Börger, Dalziel, & Fryxell, 2008). These methods, in conjunction with parallel advances in approaches using social network theory to investigate host**–**host contact (Hirsch et al., 2013; Reynolds et al., 2015), offer promise to further elucidate the interacting effects of home range size and host contact structure on disease dynamics and ORV effectiveness, in support of optimizing vaccination strategies for elimination of zoonotic diseases like rabies.

## AUTHORS’ CONTRIBUTIONS

K.M.P., A.T.G. and E.E.R. conceived the ideas and designed methodology; K.M.P. wrote the model code and conducted the simulations; K.M.P. and K.M.M. analysed the model results; and K.M.M. wrote the first draft of the manuscript. All authors provided critical feedback on the draft and gave final approval for publication.

## Data Availability

Model code available from the Dryad Digital Repository: https://doi.org/10.5061/dryad.79cnp5hrn (McClure, Gilbert, Chipman, Rees, & Pepin, 2019).
