# Supplementary material for: Variation in host home range size decreases rabies vaccination effectiveness by increasing the spatial spread of rabies virus
Source: J Anim Ecol. 2020 Feb 15;89(6):1375–86. doi: 10.1111/1365-2656.13176 (PMC7317853; doi:10.1111/1365-2656.13176)
Supplement: Supplementary file 1 [file JANE-89-1375-s001.pdf]

SUPPLEMENTAL INFORMATION

"Variation in host home range size decreases rabies vaccination effectiveness in host populations by increasing the spatial spread of rabies virus"

Katherine M. McClure[1,2,6], Amy T. Gilbert[1], Richard B. Chipman[3], Erin Rees[4, 5], Kim M. Pepin[1]

[1] United States Department of Agriculture, Animal and Plant Health Inspection Service, Wildlife Services, National Wildlife Research Center, Fort Collins, Colorado, USA
[2] Colorado State University, Department of Microbiology, Immunology, and Pathology, Fort Collins, Colorado, USA
[3] United States Department of Agriculture, Animal and Plant Health Inspection Service, Wildlife Services, National Rabies Management Program, Concord, New Hampshire, USA
[4] Land and Sea Systems Analysis Inc., Granby, Québec, Canada
[5] Public Health Risk Sciences Division, National Microbiology Laboratory, Public Health Agency of Canada, Saint-Hyacinthe, Québec, Canada

[6]Corresponding author: kat.m.mcclure@gmail.com

I. SUPPLEMENTAL TABLE AND FIGURES

Table S1. Demographic, movement, and disease parameters used in the vaccination and sensitivity simulations. Distributions: EXP = exponential, NORM = normal, WEIBULL = Weibull, GAM = gamma, POI = poisson.

**Parameters**	**Values**	**References**
**Demographic and movement**		
Longevity (number of years before natural death occurs)	~EXP(λ), λ = 0.3; mean = 3 years	(S. D. Gehrt & Prange, 2007; Johnson, 1970; Nowak, 1999; Prange, Gehrt, & Wiggers, 2003)
Age at reproductive maturity (minimum age females will conceive)	13 months	(Lotze & Anderson, 1979)
Annual conception probability (by age class of females)	0.93 (≥ 1.5 years),<br>0.54 (< 1.5 years)	(Prange et al., 2003)
Litter size (number of offspring per litter)	~ NORM(μ, σ^2); μ =4 kits , σ^2= 1	(Fritzell, Hubert Jr, Meyen, & Sanderson, 1985; Ritke, 1990; G. C. Sanderson & Hubert, 1981)
Gestation period	9 weeks	(G. Sanderson & Nalbandov, 1973)
Maximum group size	10 individuals	expert opinion
Grid-cell carrying capacity	15 raccoons/km^2	(Kennedy, Nelson, Weckerly, & Sugg, 1991; Moore & Kennedy, 1985; Sonenshine & Winslow, 1972; Urban, 1970)
Age that male dyads dissolve and males become independent	1.5 years	(S. Gehrt & Fritzell, 1998a); expert opinion
Dispersal age of males from the group	~POI(λ), λ = 10 months	(S. Gehrt & Fritzell, 1998b)
Dispersal distance (males for natal dispersal, both males and females for oversized groups)	~WEIBULL(λ, k), λ = 1.5, k = 0.3<br>median= 0.44 km, mean= 13.6 km<br>variance = 5527	(Cullingham et al., 2008; Dharmarajan, Beasley, Fike, & Rhodes, 2009)
Weekly home range radius in km (relative to home range centroid)	~GAM(k, θ), 1) k= 4.1, θ= 0.2, 2) k= 2, θ= 0.25	USDA-APHIS Wildlife Services, unpublished data
**Disease**		
Incubation period	~POI(λ), λ = 4 weeks	(Rees, Pond, Phillips, & Murray, 2008; Tinline, Rosatte, & MacInnes, 2002)
Infectious period	1 week	(Hanlon, Niezgoda, & Rupprecht, 2007)
Recovery rate/probability	0.1	expert opinion
Probability of disease-induced mortality	1	(Hanlon et al., 2007; Rupprecht et al., 1986)
Within-group transmission probability	0.5	NA
Between-group transmission probability	0.001 - 0.5	NA

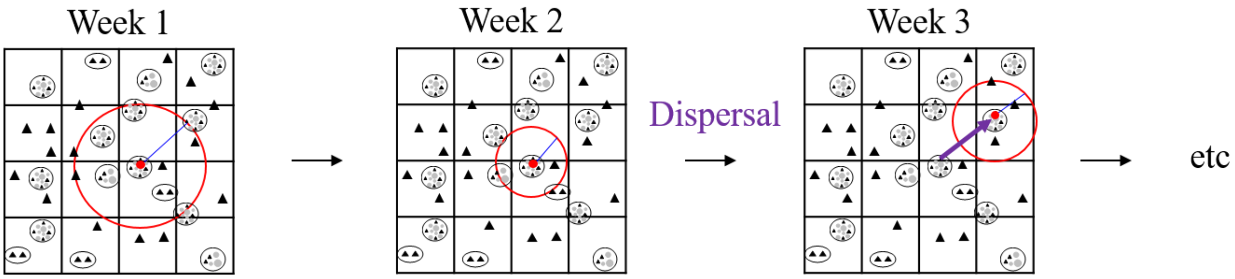

Figure S1. Host home range and contact structure. Schematic representing weekly home range size and resulting host-host contact within the gridded simulated landscape. Filled triangles are males, filled circles are females, and family groups and male dyads are enclosed by black circles. Individuals move a weekly-varying distance (the radius of the circular home range; shown by a blue line) that originates from the family group home centroid and is drawn from a gamma distribution. The red circle represents the home range explored by one individual host on a particular week. Natal dispersal (shown in purple) represents longer distance movements undertaken primarily by males, with dispersal distance drawn from a Weibull distribution. Contact among infectious and susceptible individuals and pathogen transmission (if applicable; see main text for details) occurs within the home range (red circle) of each susceptible individual at every weekly time step.

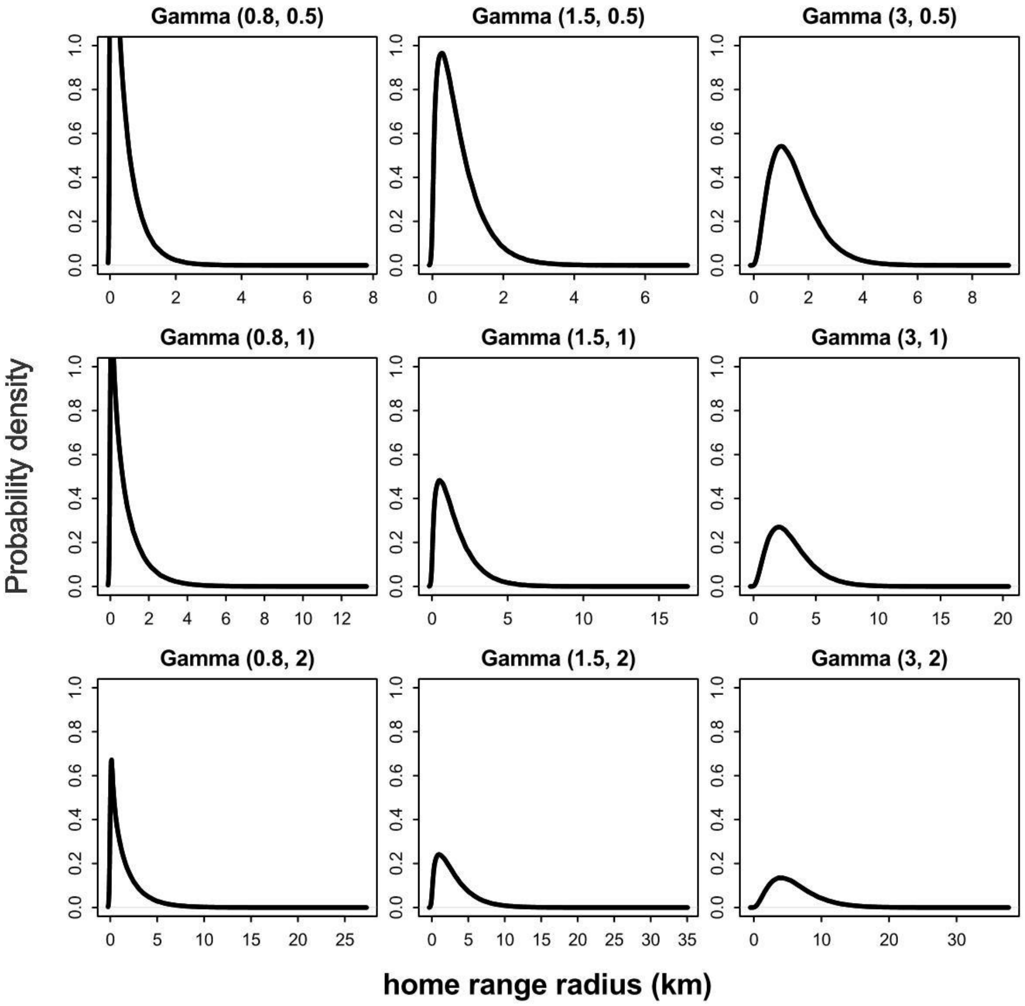

Figure S2. Gamma probability density functions. Example probability density functions for gamma distributions over the range of parameters used in the simulations with variable weekly home range radius. At each time step, each susceptible individual was assigned a home range radius drawn from a particular gamma distribution parameterized with a shape and scale parameter (Gamma (shape, scale)).

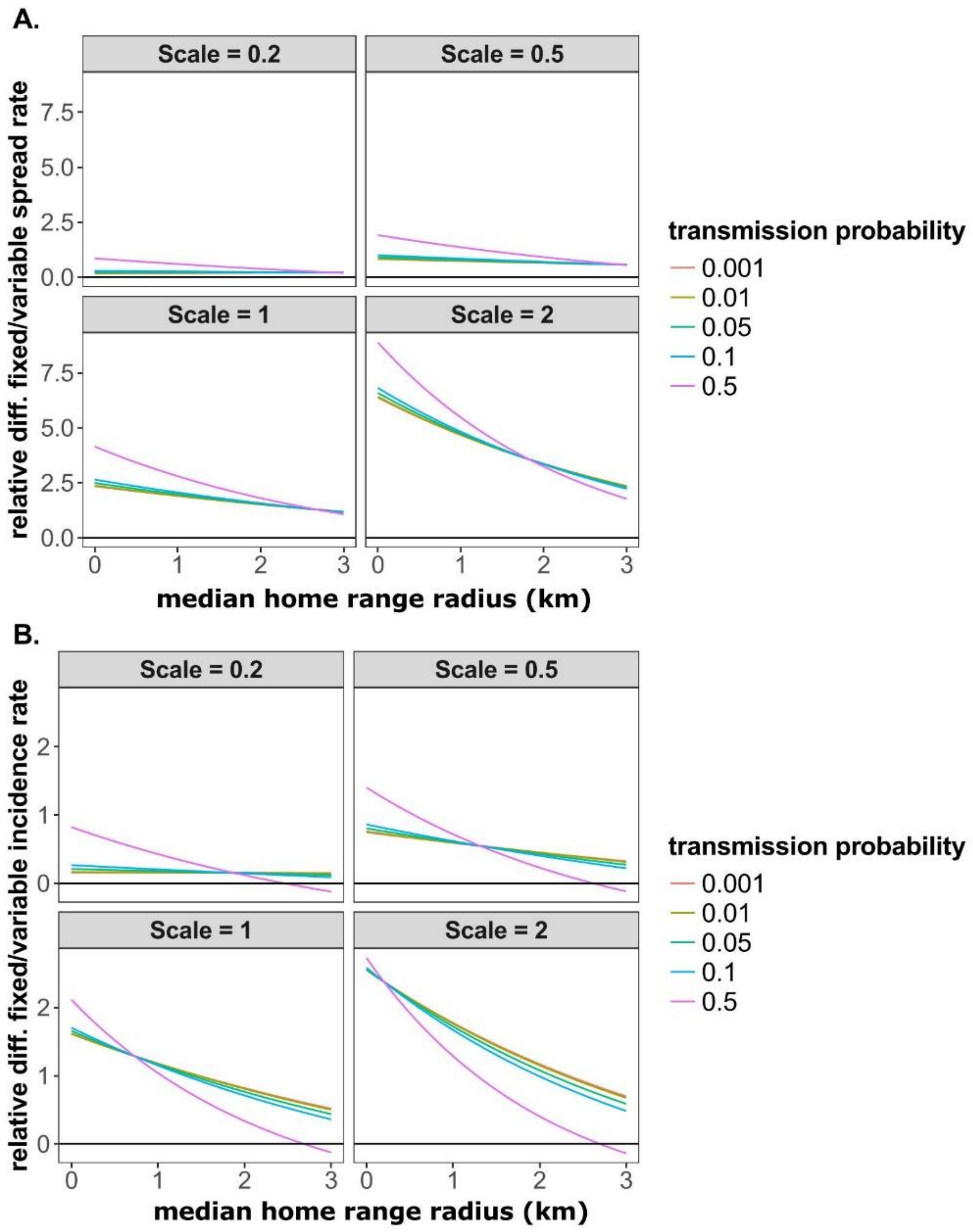

Figure S3. Relative difference of fixed vs. variable home range size in spatial spread and incidence rates. A) Relative difference in predicted spatial spread rates (km/year) between fixed and variable home range size simulations plotted against the median home range radius (in km) of the gamma-distributed weekly home range radius random variable. B) Relative difference in predicted incidence rates (annual new cases/annual maximum population size) between fixed and variable home range size simulations plotted against the median home range radius of the gamma-distributed weekly home range radius random variable. Relative differences were calculated as the difference between the predicted variable home range simulation response and the predicted fixed home range simulation response, divided by the predicted fixed home range simulation response. evaluated where the median

home range radius of the gamma distribution = fixed home range radius in km. Scale refers to the scale parameter of the gamma distribution implemented in the simulations, reflecting the variance of the distribution, with increasing values reflecting increases in home range radius variation. The black line along 0 indicates no difference between fixed and variable conditions. Values above 0 indicate the relative increase in response variables due to variation in the home range radius distribution.

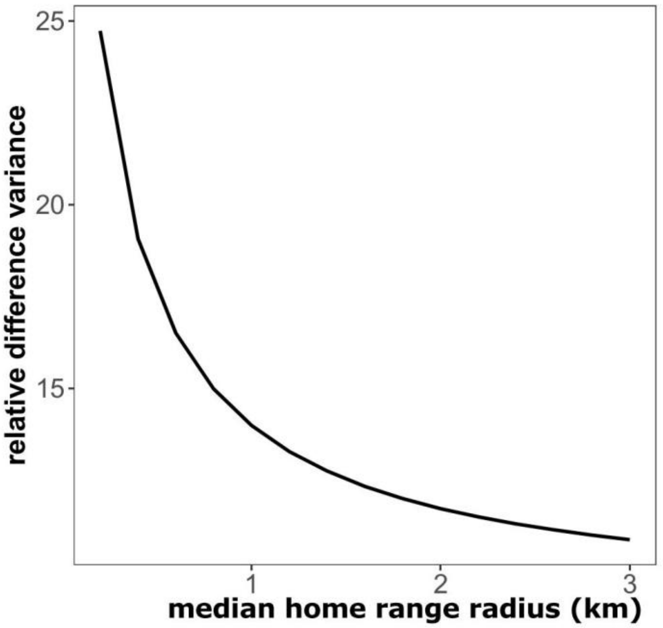

Figure S4. Relative difference in variance of high and low variance home range radius distributions used in simulations. Relative difference in the weekly host home range distribution variance between simulations with the highest variance (where the scale parameter of the gamma distribution = 2) vs. the lowest variance (where the scale parameter of the gamma distribution = 0.2) plotted against the median home range radius (km) of the weekly home range radius gamma distribution. Relative difference was calculated as the difference in the variance of the gamma distribution in high and low variance home range radius distributions divided by the variance in the low variance home range radius distribution. This shows that at lower median home range radii, the variance associated with the more variable home range radius distributions is higher relative to less variable home range radius distributions, but this difference diminishes as median home range radii increases.

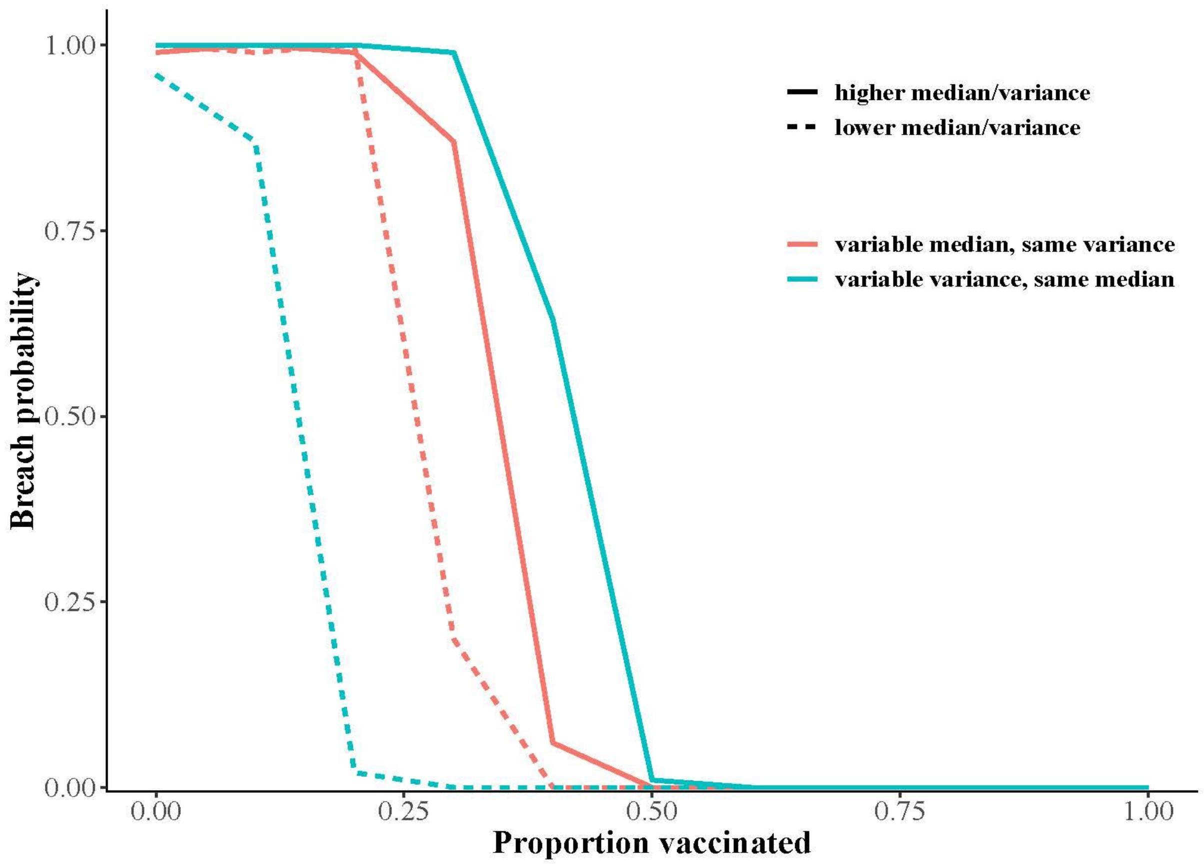

Figure S5. Breach probability given different weekly movement distributions. Rabies breach probability plotted against vaccination coverage for vaccination simulations. Line color represents weekly home range radius gamma distributions with variable medians (0.75km, 0.87km) but similar variance (0.3), or variable variance (0.49, 0.17) but similar medians (0.8km). Line type indicates higher and lower values for median or variance. Breach probability is defined as the proportion of 100 replicate simulations in which the vaccination zone was breached.

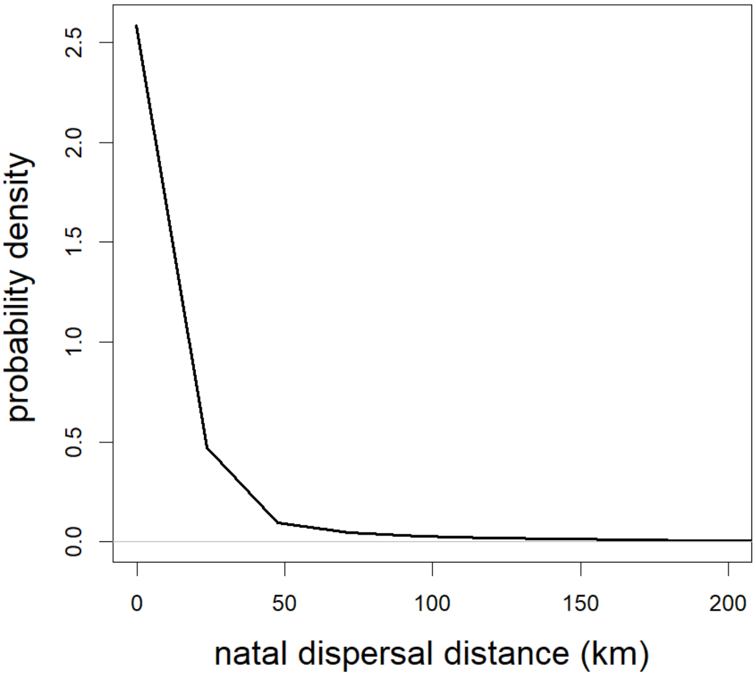

Figure S6. Weibull probability density function. Dispersal distances (in km) related to male-biased natal dispersal and dispersal to avoid over-crowding were modeled as a Weibull random variable. Dispersal distances were drawn from a Weibull distribution (shown here) that was parameterized with a scale and shape parameter (Weibull(λ, k), λ = 1.5, k = 0.3), and with a median and mean dispersal distance of 0.44 km and 13.6 km, respectively, and a variance of 5527.

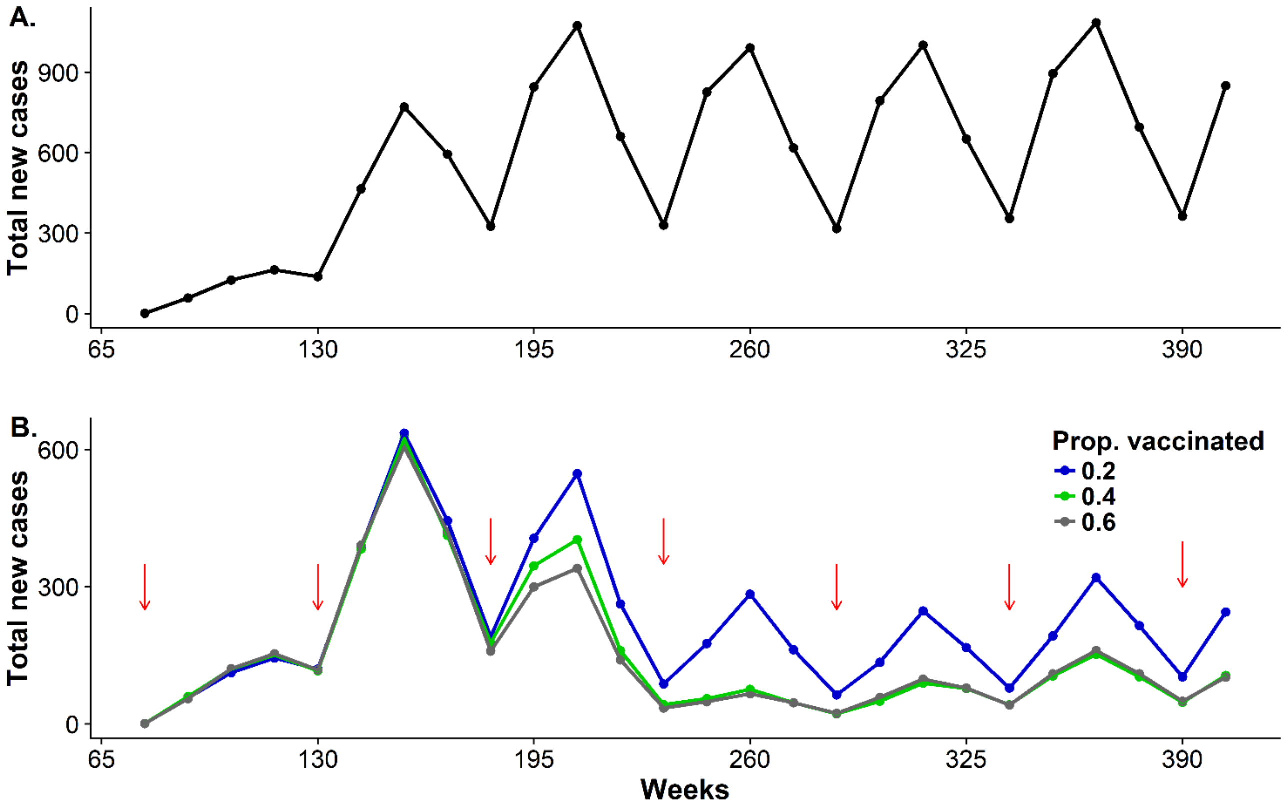

Figure S7. Total cases with and without vaccination. A) Disease dynamics in the absence of vaccination. Points are the mean total new cases at a quarterly time step (13 weeks per time step), estimated as the average of 100 simulations with the same parameter values. **B)** Points are mean total new cases for three vaccination coverage scenarios (0.2, 0.4, and 0.6 of population vaccinated). Red arrows are the quarterly time step in which vaccines were deployed (fall vaccination shown here). In both A and B, the simulated vaccination zone width was 40km, between-group transmission probability was 0.05, and weekly home range radius distribution was a gamma distribution where shape = 4.1 and scale = 0.2. Standard deviation was excluded to more easily visualize patterns.

II. STATISTICAL ANALYSES RESULTS

A. Statistical analysis of sensitivity simulations

Response variables:

Annual spatial spread rate (km/year); continuous (annual linear distance traveled per year during which disease was present at an incidence rate ≥ 0.001)

Persistence probability; binary (whether or not there were rabies cases in the last time step of simulation)

Annual incidence rate; continuous (average annual number of new rabies cases/annual maximum population size across years in which disease was present)

Main effects:

Variable/Factor	Levels
**Fixed distance moved/median of movement distribution**	0.2 – 3 km in 0.2 increments
**Shape parameter of gamma movement distribution**	0.2, 0.5, 1, 2
**Between-group transmission probability**	0.001 to 0.5 in logarithmic intervals

Table S2. Results of AIC analysis for generalized linear models of: 1) annual spatial spread rate and 2) annual incidence rate, both with a gamma distribution and a log link function, and 3) persistence probability with a binomial distribution and a logit link function. Results shown for simulations with both constant and stochastic home range radius (in km). Main effects were included in all models where 2-way interactions are indicated. K is the number of parameters. Aikaike weights were calculated to provide a measure of model selection uncertainty among fitted models (Burnham & Anderson, 2002). Asterisk indicates model with an Aikaike weight of 1.

Response variable	Home range model	Model specification	K	log likelihood	AICc	Delta AIC
**Spatial spread rate**	**Fixed**	*fixed distance moved x trans. prob.	4	-75743.38	151496.8	0
		fixed distance moved + trans. prob.	3	-75784.53	151577.1	80.30725
		Transmission probability	2	-82282.08	164570.2	13073.39249
		fixed distance moved	2	-79320.55	158647.1	7150.34216
		Intercept only	1	-83737.75	167479.5	15982.74677
	**Variable**	*All main effects and 2-way interactions	13	-392476.7	784981.5	0
		Median x scale + trans. prob	9	-392533.1	785086.2	104.726
		Scale x trans. prob. + median	9	-394437.8	788895.6	3914.16
		Median x trans. prob + scale	7	-394441.8	788899.6	3918.158
		Trans. prob x scale	8	-415863.4	831744.8	46763.375
		Median x trans. prob	4	-409855	819720.1	34738.633
		Median x scale	8	-408415.6	816849.3	31867.846
		Median + scale + trans. prob	6	-394445.7	788905.4	3923.94
		Scale + trans. prob	5	-415867.3	831746.5	46765.097
		Median + trans. prob	3	-409856.6	819721.2	34739.785
		Median + scale	5	-409603.1	819218.2	34236.76
		Scale	4	-423523	847056	62074.541
		Transmission probability	2	-422776.9	845559.8	60578.37
		Median	2	-419990.6	839987.3	55005.841
		Intercept only	1	-429034.1	858072.3	73090.832
**Incidence rate**	**Fixed**	*fixed distance moved x trans. prob.	4	-11247.86	22503.72	0
		fixed distance moved + trans. prob.	3	-11903.1	23812.2	1308.479
		Transmission probability	2	-12755.8	25515.6	3011.876
		fixed distance moved	2	-12586.91	25177.83	2674.105

		Intercept only	1	-13404.09	26810.18	4306.461
	**Variable**	*All main effects and 2-way interactions	13	-50154.35	100334.7	0
		Median x scale + trans. prob	9	-58006.74	116031.5	15696.787
		Scale x trans. prob. + median	7	-51887.8	103789.6	3454.898
		Median x trans. prob + scale	9	-57490.31	114998.6	14663.932
		Trans. prob x scale	8	-58071.49	116159	15824.283
		Median x trans. prob	4	-51993.64	103995.3	3660.592
		Median x scale	8	-59269.4	118554.8	18220.103
		Median + scale + trans. prob	6	-58307.5	116627	16292.308
		Scale + trans. prob	5	-58372.04	116754.1	16419.378
		Median + trans. prob	3	-58401.53	116809.1	16474.359
		Median + scale	5	-60062.01	120134	19799.322
		Scale	4	-60124.56	120257.1	19922.431
		Transmission probability	2	-58465.93	116935.9	16601.165
		Median	2	-60153.16	120310.3	19975.621
		Intercept only	1	-60215.59	120433.2	20098.494
**Persistence prob.**	**Fixed**	*fixed distance moved x trans. prob.	4	4297.39	-8584.777	0
		fixed distance moved + trans. prob.	3	4236.4	-8464.797	119.9794
		trans. prob	2	2498.8	-4991.599	3593.1779
		fixed distance moved	2	3359.685	-6713.369	1871.4074
		Intercept only	1	2123.488	-4242.976	4341.8009
	**Variable**	*All main effects and 2-way interactions	13	-1527.572	3083.148	0
		Median x scale + trans. prob	9	-1895.851	3811.706	104.726
		Median x trans. prob + scale	9	-2061.625	4139.252	3914.16
		Scale x trans. prob. + median	7	-2287.333	4594.669	3918.158
		Trans. prob + scale	5	-7325.597	14669.196	46763.375
		Median x trans. prob	4	-2837.068	5684.137	34738.633
		Median x scale	8	-4339.501	8697.004	31867.846
		Median + scale + trans. prob	6	-2306.647	4627.295	3923.94
		Scale + trans. prob	5	-7333.577	14679.155	46765.097
		Median + trans. prob	3	-3048.917	6105.834	34739.785
		Median + scale	5	-4613.832	9239.666	34236.76
		Scale	4	-8523.421	17056.843	62074.541
		Trans. prob	2	-7599.468	15204.937	60578.37
		Median	2	-5138.863	10283.727	55005.841
		Intercept only	1	-8737.917	17479.835	73090.832

Table S3. Parameter estimation of best-supported annual spatial spread rate model using Akaike Information Criterion (AIC; 2 points). Analyses support Figure 3A-D.

Fixed distance moved	Estimate	SE	t value	P value
Intercept	-13.376	0.506	-26.432	<0.0001
Fixed distance moved	15.077	0.156	96.710	<0.0001
Transmission probability	3.170	2.347	1.351	0.18
Trans. prob. x distance	43.327	0.772	56.087	<0.0001

n = number observations = 9,167

K= number of parameters = 4

$R^2 = 0.537$

Stochastic distance moved	Estimate	SE	t value	P value
Intercept	1.845	0.011	167.154	<0.0001
Median of gamma distribution	0.545	0.003	160.637	<0.0001
Scale 0.5	0.442	0.014	30.634	<0.0001
Scale 1	1.051	0.014	75.159	<0.0001
Scale 2	1.842	0.014	135.424	<0.0001
Transmission probability	2.837	0.036	79.584	<0.0001
Median x scale 0.5	-0.062	0.004	-14.060	<0.0001
Median x scale 1	-0.155	0.004	-35.675	<0.0001
Median x scale 2	-0.275	0.004	-64.599	<0.0001
Median x trans. prob.	-0.066	0.009	-7.039	<0.0001
Scale 0.5 x trans. prob.	0.019	0.036	0.523	0.601
Scale 1 x trans. prob.	-0.060	0.036	-1.660	0.097
Scale 2 x trans. prob.	-0.336	0.036	-9.337	<0.0001

n = number observations = 46,402

K= number of parameters = 13

$R^2 = 0.59$

Table S4. Parameter estimation of best-supported pathogen persistence probability model using Akaike Information Criterion (AIC; 2 points). Analyses support Figure 3E-H.

**Fixed distance moved**	**Estimate**	**SE**	**t value**	**P value**
Intercept	-2.573	0.043	-59.196	<0.0001
Fixed distance moved	0.724	0.014	50.085	<0.0001
Transmission probability	5.600	0.302	18.529	<0.0001
Trans. prob. x distance	-7.449	0.220	-33.911	<0.0001

n = number observations = 15,000
K = number of parameters = 4
$R^2 = 0.184$

**Stochastic distance moved**	**Estimate**	**SE**	**t value**	**P value**
Intercept	-2.087	0.038	-55.345	<0.0001
Median of gamma distribution	0.697	0.013	54.587	<0.0001
Transmission probability	14.342	0.284	50.572	<0.0001
Scale 0.5	0.719	0.050	14.386	<0.0001
Scale 1	1.523	0.049	31.148	<0.0001
Scale 2	2.176	0.049	44.022	<0.0001
Median x trans. prob.	-13.993	0.168	-83.305	<0.0001
Scale 0.5 x trans. prob.	2.581	0.321	8.032	<0.0001
Scale 1 x trans. prob.	-0.332	0.322	-1.031	0.303
Scale 2 x trans. prob.	-10.371	0.352	-29.447	<0.0001
Median x scale 0.5	-0.235	0.017	-14.100	<0.0001
Median x scale 1	-0.505	0.016	-30.597	<0.0001
Median x scale 2	-0.752	0.017	-44.557	<0.0001

n = number observations = 60,000
K = number of parameters = 13
$R^2 = 0.186$

Table S5. Parameter estimation of best-supported annual incidence rate model using Akaike Information Criterion (AIC; 2 points). Analyses support Figure 3I-L.

Fixed distance moved	Estimate	SE	t value	P value
Intercept	-3.044	0.018	169.934	<0.0001
Fixed distance moved	0.554	0.006	98.856	<0.0001
Transmission probability	3.947	0.086	45.991	<0.0001
Trans. prob. x distance	-0.582	0.029	-20.391	<0.0001

n = number observations = 9,732

K = number of parameters = 4

R^2 = 0.34

Stochastic distance moved	Estimate	SE	t value	P value
Intercept	-2.716	0.012	-227.10	<0.0001
Median of gamma distribution	0.489	0.004	131.264	<0.0001
Scale 0.5	0.414	0.016	26.099	<0.0001
Scale 1	0.815	0.015	52.872	<0.0001
Scale 2	1.122	0.015	74.761	<0.0001
Transmission probability	3.352	0.040	83.465	<0.0001
Median x scale 0.5	-0.091	0.005	-18.434	<0.0001
Median x scale 1	-0.179	0.005	-37.039	<0.0001
Median x scale 2	-0.244	0.005	-51.462	<0.0001
Median x trans. prob.	-0.485	0.011	-46.000	<0.0001
Scale 0.5 x trans. prob.	-0.274	0.041	-6.670	<0.0001
Scale 1 x trans. prob.	-0.559	0.041	-13.626	<0.0001
Scale 2 x trans. prob.	-0.813	0.041	-19.912	<0.0001

n = number observations = 47,378

K = number of parameters = 13

R^2 = 0.3

B. Statistical analysis of vaccination simulations

Response:
Binary (whether or not rabies breached the vaccination zone)

Main effects:

Variable/Factor	Levels
**Vaccination deployment timing**	Fall, spring, fall and spring
**Proportion of population immunized**	0 to 100% in 0.1 increments
**Width of vaccination zone**	20km^2 , 40km^2 , and 60km^2
**Weekly movement**	Gamma(shape= 4.1, scale = 0.2)<br>Gamma(shape=2, scale= 0.5)
**Between-group transmission probability**	0.5 and 0.1

Table S6. Results of AIC analysis for generalized linear models of vaccination zone breach probability with a binomial distribution and a logit link function for vaccination simulations. Main effects were included in all models where 2-way interactions are indicated. K is the number of parameters in the model. The top ten best-supported models are shown. Aikaike weights were calculated to provide a measure of model selection uncertainty among fitted models (Burnham & Anderson, 2002). Asterisk indicates model with an Aikaike weight of 1.

Model specification	K	log likelihood	AICc	Delta AIC	AIC weight
All main effects and 2-way interactions	27	-3700.075	7454.188	0	1
All interactions except prop. vaccinated: trans. prob.	26	-3722.473	7496.982	42.79354	0
All interactions except timing x trans. prob.	25	-3728.677	7507.387	53.19927	0
All interactions except prop. vaccinated x trans. prob. & timing x trans. prob.	24	-3730.56	7509.151	54.96322	0
All interactions except area x trans. prob.	25	-3732.351	7514.735	60.54698	0
All interactions except area x trans. prob. & timing x trans. prob.	24	-3738.66	7525.351	71.16281	0
All interactions except prop. vaccinated x trans. prob. & timing x trans. prob. & areas x trans. prob.	22	-3747	7538.026	83.83756	0
All interactions except area x trans. prob. & timing x trans. prob.	23	-3746.999	7540.026	85.83786	0
All interactions except area x timing	23	-3753.471	7552.971	98.78287	0
All interactions except area x trans. prob. & area x timing	21	-3759.986	7561.995	107.8067	0

Table S7. Parameter estimation of the best-supported model of breach probability using Akaike Information Criterion (AIC; 2 points), which includes all main effects and all possible two-way interactions. Simulation data was fit to generalized linear models with a binomial distribution and a logit link. Analyses support Figure 5.

Model term	Estimate	SE	t value	P value
Intercept	3.23266	0.18231	17.732	<0.0001
shape_2	10.17199	0.37365	27.223	<0.0001
transmission probability_0.1	9.40278	0.3173	29.634	<0.0001
proportion vaccinated	-45.7251	1.14096	-40.076	<0.0001
timing_spring	-1.3584	0.21207	-6.405	<0.0001
timing_spring+fall	-2.32445	0.20602	-11.283	<0.0001
barrier area_20km	4.70812	0.23768	19.808	<0.0001
barrier area_40km	2.18564	0.19935	10.964	<0.0001
shape_2 x trans. prob._0.1	-5.14293	0.27737	-18.542	<0.0001
shape_2 x prop. vaccinated	13.78053	0.64275	21.44	<0.0001
shape_2 x timing_spring	-5.15968	0.42305	-12.196	<0.0001
shape_2 x timing_spring+fall	1.85079	0.44156	4.191	<0.0001
shape_2 x area_20km	-4.31862	0.28276	-15.273	<0.0001
shape_2 x area_40km	-2.30984	0.28159	-8.203	<0.0001
trans. prob._0.1 x prop vaccinated	3.98704	0.56487	7.058	<0.0001
trans. prob._0.1 x timing_spring	-2.38452	0.32819	-7.266	<0.0001
trans. prob._0.1 x timing_spring+fall	0.49002	0.31139	1.574	0.116
trans. prob._0.1 x area_20km	-2.02252	0.2496	-8.103	<0.0001
trans. prob._0.1 x area_40km	-0.66244	0.24733	-2.678	0.007
prop. vaccinated x timing_spring	27.19396	0.90888	29.92	<0.0001
prop. vaccinated x timing_spring+fall	-4.81453	1.29356	-3.722	0.0002
prop. vaccinated x area_20km	7.25877	0.61762	11.753	<0.0001
prop. vaccinated x area_40km	2.563	0.61626	4.159	<0.0001
timing_spring x area_20km	-2.31549	0.26872	-8.617	<0.0001
timing_spring+fall x area_20km	0.65203	0.25932	2.514	0.012
timing_spring x area_40km	-0.3702	0.25898	-1.429	0.153
timing_spring+fall x area_40km	0.02349	0.20746	0.113	0.901
	n= observations = 39,600			
	K = number of parameters = 27			
	R^2 = 0.91			

III. SUPPLEMENTAL METHODS

We describe our modeling approach here using the updated Overview, Design Concepts, and Details protocol for individual-based models (Grimm et al., 2006, 2010).

A. Overview

i. Purpose

Our main objectives were to 1) investigate the effect of variation of host home range and home range space use on rabies epidemiology in a wild carnivore population, 2) explore the effect of wildlife host home range size and home range space use on the probability that rabies virus will breach an oral rabies vaccination (ORV) zone, and 3) identify the relative effectiveness of feasible components of ORV strategies while accounting for realistic ecological processes in host demography and rabies epidemiology. The components of ORV strategies that we investigated included: seasonal timing of ORV application, width of the vaccination zone, and fraction of wild animals immunized. We first conducted a sensitivity analysis by varying weekly host home range size distributions and disease transmission probabilities to explore the effect of variable movement on epidemiological processes. We then examined the effects of host home range size variation on ORV effectiveness by comparing epidemiological outputs from two realistic host home range radius distributions with different variances but similar magnitudes of host home range size, using a full factorial design on the three ORV strategy components.

ii. Entities, state variables, and scales

We modeled raccoon individuals as entities with the following attributes: age, sex, group, and individual raccoon identification (ID), natal dispersal age and status, litter size, reproductive status, longevity, home range centroid (x, y coordinates), and grid cell ID. Epidemiological state variables included susceptible, exposed, infectious, and recovered disease classes. Each grid cell represented 1km^2. The total area of the modeled landscape ranged between 820km^2 and 1620 km^2 depending on the width of the vaccination zone (20, 40, and 60 km, Figs. 1A & 2). Each cell had a carrying capacity of 15 individuals, and was based on published raccoon densities from suburban habitats (Urban 1970; Sonenshine and Winslow 1972; Moore and Kennedy 1985, Table 1). Each raccoon family group had a maximum of 10 individuals. Individuals in the same family group were assigned the same grid cell ID and home range centroid point from which movement originated. Sex and natural longevity were assigned at birth, while all other states changed depending on age, sex, population density, or exposure to an infectious individual.

B. Process overview and scheduling

Models were updated on a weekly time step. We ran ten-year vaccination simulations and eight-year sensitivity simulations. In each simulation, we initiated disease by transitioning all individuals in the middle cell of the seeding zone during week 10, following a one-year period for

demographic burn-in (i.e., seeding rabies virus in week 63 of the simulation). During the post-burn-in simulation, the order and processes per time step were:

- Update ages and reproductive clocks
- Weekly home range size and disease state transitions: randomly assign each individual a weekly home range radius relative to their home range centroid. Transition susceptible individuals to exposed class based on within or between group transmission probabilities given the infectious individuals in their home range at week *t* (see Equation 1 below). Update disease states of exposed and infectious individuals, if applicable.
- Vaccination: randomly select a fixed proportion of the population within the ORV zone, and update their disease state to recovered.
- Natural mortality: remove individuals that reach longevity age.
- Natal dispersal: change the home range centroid and grid cell ID (if applicable) of dispersal-age males that group together and dispersal-age males that become solitary (procedure described below). Update natal dispersal status to complete.
- Social dynamics: for family groups that become too large (i.e., greater than 10 individuals), disperse half to another home range centroid and grid cell ID (if applicable) using the same procedure as natal dispersal.
- Density-dependent mortality: for grid cells that exceed the fixed carrying capacity, remove youngest individuals.
- Conception: identify new conceptions, initialize gestation clocks, choose litter size, turn-off postnatal clocks.
- Births: when gestation is complete, assign attributes to each new litter (size determined at random from normal distribution, Table 1). Assign sex in 1:1 ratio, assign the grid cell ID, group ID and home range centroid as mother; assign other individual-level factors.

C. Design concepts

i. Basic principles

We explicitly modeled raccoon population dynamics and RABV transmission on the landscape in two separate analyses. In a first set of simulations (sensitivity analysis), we investigated the effect of variable home range size on disease processes by varying weekly host home range radius distributions and disease transmission probabilities in the absence of vaccination. In the second set of simulations (vaccination analysis), we explored how three components of ORV strategies— proportion of the population vaccinated, timing of ORV, and width of the ORV zone (Fig. 2)—affected vaccination effectiveness. We also examined how data-informed weekly home range size affected ORV zone breach probability because raccoons exhibit variation in home range sizes across different habitats (Beasley & Rhodes, 2010; Šálek, Drahníková, & Tkadlec, 2015).

ii. Emergence

Natal dispersal and overcrowding dispersal distances were defined by a random distribution with set parameters, but the realized dispersal distance emerged from both the random distribution and the spatial distribution of current population density. Model algorithms forced individuals to move twice as far as the randomly drawn dispersal distance if the grid into which the individual was moving was already at the grid-level carrying capacity. New distances were considered until either an individual dispersed off the grid permanently or found a grid cell with space.

iii. Sensing and Interaction

Raccoons were capable of sensing social group dynamics, within group density, and grid-level density. Susceptible and infectious individuals interacted to determine disease transmission. We did not incorporate explicit interaction of wildlife managers and raccoons.

iv. Stochasticity

Longevity, weekly movement distance, dispersal distances, dispersal age (males only), litter size, and disease incubation period were random distributions.

v. Collectives

Raccoons were born into family groups. Males remained within family groups until natal dispersal. Females remained in family groups until group size exceeded the group carrying capacity, if applicable.

vi. Observation

For the sensitivity simulations, total cases by sex and age, total population size, and the linear distance the pathogen moved were recorded on a weekly timescale. For the vaccination simulations, the same observations were recorded at a quarterly time step, as well as immunity by age class and the total cases within the vaccination zone.

D. Initialization

The simulated landscape was initialized with raccoons equal to the carrying capacity of each cell. Sex was randomly assigned in a 1:1 ratio. Longevity and age were assigned randomly. We initialized group structure by grouping females that were the minimum reproductive age or older with individuals that were younger than the minimum dispersal age of males. Individual and group IDs were assigned at random, ensuring that there was at least one reproductive female

per group and that the total number per group did not exceed the group carrying capacity. Home range centroid coordinates for family groups and males aged 1.5 years and older were randomly chosen within that grid cell. All individuals were assigned initially to the susceptible disease class. Prior to disease introduction, 10% of individuals of sufficient age were randomly chosen and transitioned to the recovered disease class to model natural rabies resistance observed in raccoon populations (Slate et al., 2009). Populations were allowed to undergo demographic dynamics for one year, after which ~15 infectious individuals were introduced into the seeding zone on week 63 of the simulation.

E. Input data

Input parameters are described in Table S1. We also input landscapes describing the grid cell IDs, locations, and zonal information. The landscape contained 4 distinct zones (Fig. 1): seeding (1 x 20 km), spreading (10 x 20 km), vaccination zone (20-60 x 20 km), and breach (10 x 20 km).

F. Submodels

i. Natural mortality

Natural mortality was modeled as a random variable (Table S1). Longevity was assigned to individuals at birth, and was drawn from an exponential distribution (values rounded to nearest week; mean = 3 years; Table S1). Individuals were permanently removed at the age of longevity. Individuals were also subject to density-dependent mortality, as well as disease-induced mortality if they became infected and infectious.

ii. Social structure

We modeled females and young in family groups. We assumed that the maximum group size was 10 (maximum of 2 adult females and 4 kits/dam). Conception probability varied by age and gestation period was 9 weeks (Table S1). Litter size was drawn from a normal distribution (values rounded to integers; mean = 4 kits; Table S1). Longevity was drawn from an exponential distribution (values rounded to nearest week; mean = 3 years; Table S1). Dispersal-age males were either part of male-only dyads or were solitary following a second dispersal event at 1.5 years of age. Raccoons exhibit variable degrees of sociality, with field and genetic studies suggesting that females are often philopatric, and daughters may associate with mothers and offspring into adulthood (Cullingham et al., 2008; Dharmarajan et al., 2009; S. Gehrt & Fritzell, 1998b). Natal dispersal is male-biased, and males may associate in relatively long-lasting, non-familial dyads followed by separation to become independent as they mature (S. D. Gehrt, Gergits, & Fritzell, 2008; S. Gehrt & Fritzell, 1998a; Hirsch, Prange, Hauver, & Gehrt, 2013).

iii. Dispersal

We modeled two types of dispersal movements: post-natal dispersal, and relocation by both males and females due to family group overcrowding (Fig. 2, Table 1).. Dispersal distance and natal dispersal age were drawn from a Weibull distribution (mean = 13.6km; Fig. S6, Table S1) and a Poisson distribution (mean = 10 months; Table S1), respectively. For dispersal due to group overcrowding (>10 individuals, Table S1), groups split into two, with one group dispersing randomly. Movement into cells at grid-level carrying capacity was not allowed. Dispersal events occurred as follows. For each 45-degree angle from the home range centroid, dispersal movement distances were chosen at random from the specified random distribution of dispersal distances. New potential x, y coordinates were calculated as x = distance × cos(angle) + current × coordinate, y = distance × sin (angle) + current y coordinate. We chose 45-degree angles to systematically search all surrounding neighbor grid cells at the randomly drawn dispersal distance (Queen's case contiguity). Dispersal occurred if the target locations were located within the simulated landscape and within a grid with fewer raccoons than the carrying capacity. When more than one location satisfied the criteria, the new location was chosen at random among those that were considered valid. If no locations were valid, the distance values were doubled, and the process repeated until a valid set of coordinates were found or the individual moved off the landscape. We thus assumed that dispersing individuals would move farther and farther out in a crowded landscape, reflecting evidence that high density populations can decrease the immigration success of some mammal species (Bowler & Benton, 2015). Individuals that traveled off the landscape were lost permanently and did not return to the landscape.

iv. Disease transmission

We modeled epidemiological dynamics of rabies with four disease states: susceptible, exposed but not infectious, infectious, and recovered (Figs. 1B & S1A), as detailed in the methods of the main text. Incubation period of exposed individuals was drawn from a Poisson distribution with a mean of 4 weeks (Tinline *et al.* 2002; Rees *et al.* 2008, Table 1). Disease-induced mortality for infectious individuals was 100% and death from disease occurred one week after individuals transitioned from the exposed class into the infectious disease class (Hanlon et al., 2007). Recovery rate of exposed individuals was 10% to capture variation in natural resistance to rabies observed in raccoons (Slate et al., 2014). Susceptible individuals that were vaccinated entered the recovered disease class with a two-week lag following the vaccination activity.

v. Vaccination

Vaccination comprised three components: coverage or proportion of animals immunized within the ORV zone, timing and frequency of vaccination, and ORV zone width (Fig. 2). We examined three zone widths: 20 km, 40 km, and 60 km, with larger widths implemented as additional grid cells in the ORV zone of the landscape. We modeled vaccination coverage by randomly selecting a fixed proportion of the population within the ORV zone, and converting susceptible individuals within this subset to the recovered disease class with a delay of 2 weeks

to account for the development of immunity in vaccinated individuals. Raccoons under 17 weeks are still nursing (Montgomery, 1969); only individuals older than 17 weeks could be vaccinated because delivery was assumed to be through oral vaccine baiting. Vaccination coverage ranged from 0 - 100% where 0% was the no vaccination control (Figs. 2 & S2). Raccoon ORV was timed to occur during fall, spring, or both fall and spring to explore different strategies for timing and frequency, because seasonality in host or pathogen ecology can drive disease dynamics such that intervention timing and frequency may influence intervention effectiveness (Altizer et al., 2006). Vaccination was assumed to be 100% effective for susceptible and exposed individuals and imparted lifetime immunity. Vaccination was assumed ineffective on infectious individuals.

vi. Density-dependent mortality

In order to limit population abundance to our given within-grid carrying capacity, we implemented density-dependent mortality. When grid cells exceeded the carrying capacity (15 individuals per km^2), individuals within the grid were randomly chosen and removed from the simulation, with younger individuals taken first to mimic observed differences in juvenile and adult survivorship (G. Gehrt & Fritzell, 1999). We assumed no time lag between when grids exceeded the within-grid carrying capacity and the resulting effects on mortality of individuals within that grid.

vii. Conceptions and births

We modeled reproduction as a single birth pulse beginning in April and extending to mid-May for a 6 week breeding period (George & Stitt, 1951; Rees et al., 2008; Stuewer, 1943). Conception probability varied by age and gestation period was 9 weeks (Table S1), thus mating occurred from mid-February to early March. The probability of conception varied with age such that females > 1.5 years of age were more likely to conceive relative to females < 1.5 years of age, implemented using scaling parameters on conception probability (Table 1). Litter size was drawn from a normal distribution (values rounded to integers; mean = 4 kits; Table S1). Litter size was a drawn from a normal distribution with a mean of 4 kits and variance = 1 (Sanderson and Hubert 1981; Fritzell *et al.* 1985; Ritke 1990; Table 1).

G. Movement data collection and fitting

We fit weekly distances to a gamma distribution and estimated the shape and scale parameters (shape= 4.1 and scale= 0.2) using maximum likelihood methods. The mean and median of the gamma distribution fit to these data was 0.82 km and 0.75 km, respectively, and the variance was the probability that any individual moved more than 3 km was 0.00001. We used a second gamma distribution with a higher mean, median, and variance (shape = 2, scale = 0.5, mean = 1 km, median = 0.84) to explore the effect of a more variable host movement distribution on vaccination effectiveness; the probability any individual moved more than 3 km was approximately 0.018—substantially higher than the less variable movement distribution. We

estimated similar shape and scale parameters from an independent study of GPS-collared raccoons in a suburban ORV area in Tennessee over the same months during 2013-2014 (Berentsen et al., 2017; data not shown). Data collection and fitting procedures are further detailed in the methods in the main text.

H. Simulations

We conducted outbreak simulations using multiple levels of three parameters: 1) shape and 2) scale parameters of the weekly host movement gamma distribution, and 3) between-group transmission probability. We focused on weekly host home range distributions rather than host dispersal movement distributions because results of a preliminary sensitivity analysis (data not shown) indicated that disease outcomes were not sensitive to variability in the natal dispersal variable but were sensitive to variability in the weekly home range size variable.

To further investigate the relative effects of magnitude (median) and variability of weekly home range radius gamma distributions on breach probability, we modeled vaccination using four additional gamma distributions. Two gamma distributions had similar medians (median = 0.8 km) but differed in variance (variance = 0.49, Gamma(shape=1.96, scale=0.5); variance = 0.17, Gamma(shape=4.3, scale=0.2). Two gamma distributions had similar variance (variance = 0.3) but differed in median (median = 0.75km, Gamma(shape=2.2, scale=0.4; median = 0.87 km, Gamma(shape=2.8, scale=0.35). Vaccination zone area (40km), ORV application time and frequency (fall only), and transmission probability (0.05) were held constant. We ran 43 unique parameter sets, with 100 ten-year replicate simulation per parameter set, for a total of 44,000 simulations. We defined vaccination effectiveness as 1 – *v*, where v is the minimum vaccination coverage required to reduce breach probability to zero.

R_0 Simulation

We calculated R_0 (the average number of infectious cases caused by one infectious individual in a completely susceptible population) using numerical simulation for one set of conditions that implemented the data-informed gamma distribution (Gamma(shape = 4.1, scale = 0.2)) for weekly home range movement and a transmission probability of 0.05. We conducted 1000 two-year replicate simulations where we tracked the number of transmissions caused by a single index case, and took the mean number of index case transmissions as R_0.

I. Model outputs and statistical analysis

See methods in main text for description of the main model outputs and statistical analyses.

IV. SUPPLEMENTAL RESULTS

A. Additional vaccination simulation

Increases in the variance of the weekly movement gamma distribution led to a 42.9% decrease in vaccination effectiveness, when median host home range radius was held constant between host home range distributions. Increases in median host home range radius led to a 16.7% decrease in effectiveness, when variance was held constant (Supporting Information Figure S5).

LITERATURE CITED

Altizer, S., Dobson, A., Hosseini, P., Hudson, P., Pascual, M., & Rohani, P. (2006). Seasonality and the dynamics of infectious diseases. *Ecology Letters*, *9*(4), 467–484. doi: 10.1111/j.1461-0248.2005.00879.x

Beasley, J. C., & Rhodes, O. E. (2010). Influence of patch- and landscape-level attributes on the movement behavior of raccoons in agriculturally fragmented landscapes. *Canadian Journal of Zoology*, *88*(2), 161–169. doi: 10.1139/Z09-137

Berentsen, A. R., Patrick, E. M., Blass, C., Wehner, K., Dunlap, B., Hicks, B., … Vercauteren, K. C. (2017). Seroconversion of raccoons following two oral rabies vaccination baiting strategies. *Journal of Wildlife Management*, *2007*, 1–6. doi: 10.1002/jwmg.21368

Bowler, D. E., & Benton, T. G. (2015). Causes and consequences of animal dispersal strategies : relating individual behaviour to spatial dynamics. *Biological Reviews*, *80*(02), 205–225. doi: 10.1017/S1464793104006645

Burnham, K., & Anderson, D. (2002). *Model selection and inference: a practical information-theoretic approach*. New York, NY: Springer-Verlag, New York.

Cullingham, C., Pond, B., Kyle, C., Rees, E., Rosatte, R., & White, B. (2008). Combining direct and indirect genetic methods to estimate dispersal for informing wildlife disease management decisions. *Molecular Ecology*, *17*(22), 4874–4886. doi: 10.1111/j.1365-294X.2008.03956.x

Dharmarajan, G., Beasley, J. C., Fike, J. A., & Rhodes, O. E. (2009). Population genetic structure of raccoons (*Procyon lotor*) inhabiting a highly fragmented landscape. *Canadian Journal of Zoology*, *87*(9), 814–824. doi: 10.1139/Z09-072

Fritzell, E. K., Hubert Jr, G. F., Meyen, B. E., & Sanderson, G. C. (1985). Age-specific reproduction in Illinois and Missouri raccoons. *Journal of Wildlife Management*, *49*(4), 901–905. doi: 10.2307/3801366

Gehrt, G., & Fritzell, E. (1999). Survivorship of a Nonharvested Raccoon Population in South Texas. *Journal of Wildlife Management*, *63*(3), 889–894.

Gehrt, S. D., Gergits, W. F., & Fritzell, E. K. (2008). Behavioral and genetic aspects of male social groups in raccoons. *Journal of Mammalogy*, *89*(6), 1473–1480. doi: 10.1644/07-MAMM-A-

403.1

Gehrt, S. D., & Prange, S. (2007). Interference competition between coyotes and raccoons: A test of the mesopredator release hypothesis. *Behavioral Ecology*, *18*(1), 204–214. doi: 10.1093/beheco/arl075

Gehrt, S., & Fritzell, E. (1998a). Duration of Familial Bonds and Dispersal Patterns for Raccoons in South Texas. *Journal of Mammalogy*, *79*(3), 859–872.

Gehrt, S., & Fritzell, E. (1998b). Resource distribution, female home range dispersion and male spatial interactions: group structure in a solitary carnivore. *Animal Behaviour*, *55*(5), 1211–1227. doi: 10.1006/anbe.1997.0657

George, J., & Stitt, M. (1951). March litters of raccoons (*Procyon lotor*). *Journal of Mammalogy*, *32*(2), 218–219.

Grimm, V., Berger, U., Bastiansen, F., Eliassen, S., Ginot, V., Giske, J., … DeAngelis, D. L. (2006). A standard protocol for describing individual-based and agent-based models. *Ecological Modelling*, *198*(1–2), 115–126. doi: 10.1016/j.ecolmodel.2006.04.023

Grimm, V., Berger, U., DeAngelis, D. L., Polhill, J. G., Giske, J., & Railsback, S. F. (2010). The ODD protocol: A review and first update. *Ecological Modelling*, *221*(23), 2760–2768. doi: 10.1016/j.ecolmodel.2010.08.019

Hanlon, C., Niezgoda, M., & Rupprecht, C. (2007). Rabies in terrestrial animals. In A. Jackson & W. Wunner (Eds.), *Rabies* (2nd ed., pp. 201–246). Cambridge, MA: Academic Press.

Hirsch, B. T., Prange, S., Hauver, S. A., & Gehrt, S. D. (2013). Raccoon Social Networks and the Potential for Disease Transmission. *PLoS ONE*, *8*(10), 4–10. doi: 10.1371/journal.pone.0075830

Johnson, A. (1970). *Biology of the raccoon (*Procyon lotor varius *Nelson and Goldman) in Alabama*. Auburn, AL: Auburn University Agricultural Experiment Station.

Kennedy, M. L., Nelson, J. P., Weckerly, F. W., & Sugg, D. W. (1991). An Assessment of Selected Forest Factors and Lake Level in Raccoon Management. *Wildlife Society Bulletin*, *19*(2), 151–154.

Lotze, J.-H., & Anderson, S. (1979). *Procyon lotor*. *Mammalian Species*, (119), 1. doi: 10.2307/3503959

Montgomery, G. (1969). Weaning of captive raccoons. *Journal of Wildlife Management*, *33*(1), 154–159.

Moore, D., & Kennedy, M. (1985). Factors Affecting Response of Raccoons to Traps and Population Size Estimation. *The American Midland Naturalist*, *114*(1), 192–197.

Nowak, R. M. (1999). *Walker's mammals of the world*. Retrieved from https://books.google.com/books/about/Walker_s_Mammals_of_the_World.html?id=T37sFCl43E8C

Prange, S., Gehrt, S., & Wiggers, E. (2003). Demographic factors contributing to high raccoon densities in urban landscapes. *Journal of Wildlife Management*, *67*(2), 324–333.

Rees, E. E., Pond, B. A., Phillips, J. R., & Murray, D. (2008). Raccoon ecology database: A resource for population dynamics modelling and meta-analysis. *Ecological Informatics*, *3*(1), 87–96. doi: 10.1016/j.ecoinf.2008.01.002

Ritke, M. E. (1990). Quantitative assessment of variation in litter size of the racoon *Procyon lotor*. *American Midland Naturalist*, *123*(2), 390–398.

Rupprecht, C. E., Wiktor, T. J., Johnston, D. H., Hamir, a N., Dietzschold, B., Wunner, W. H., … Koprowski, H. (1986). Oral immunization and protection of raccoons (*Procyon lotor*) with a vaccinia-rabies glycoprotein recombinant virus vaccine. *Proceedings of the National Academy of Sciences of the United States of America*, *83*(20), 7947–7950. doi: 10.1073/pnas.83.20.7947

Šálek, M., Drahníková, L., & Tkadlec, E. (2015). Changes in home range sizes and population densities of carnivore species along the natural to urban habitat gradient. *Mammal Review*, *45*(1), 1–14. doi: 10.1111/mam.12027

Sanderson, G. C., & Hubert, G. (1981). Selected demographic characteristics of Illinois raccoons (*Procyon lotor*). In J. Chapman & D. Pursley (Eds.), *Proceedings Worldwide Furbearers Conference* (pp. 487–513). Frostburg, MD.

Sanderson, G., & Nalbandov, A. A. V. A. (1973). The reproductive cycle of the raccoon in Illinois. *Illinois Natural History Survey Bulletin*, *31*(2). Retrieved from https://www.ideals.illinois.edu/handle/2142/44058

Slate, D., Algeo, T. P., Nelson, K. M., Chipman, R. B., Donovan, D., Blanton, J. D., … Rupprecht, C. E. (2009). Oral rabies vaccination in North America: Opportunities, complexities, and challenges. *PLoS Neglected Tropical Diseases*, *3*(12), 1–9. doi: 10.1371/journal.pntd.0000549

Slate, D., Chipman, R. B., Algeo, T. P., Mills, S. A., Nelson, K. M., Croson, C. K., … Rupprecht, C. E. (2014). Safety and immunogenicity of Ontario Rabies Vaccine Bait (ONRAB) in the first US field trial in raccoons (*Procyon lotor*). *Journal of Wildlife Diseases*, *50*(3), 582–595. doi: 10.7589/2013-08-207

Sonenshine, D., & Winslow, E. (1972). Contrasts in Distribution of Raccoons in Two Virginia Localities. *Journal of Wildlife Management*, *36*(3), 838–847.

Stuewer, F. W. (1943). Raccoons: Their Habits and Management in Michigan. *Ecological Monographs*, *13*(2), 203–257. doi: 10.2307/1943528

Tinline, R., Rosatte, R., & MacInnes, C. (2002). Estimating the incubation period of raccoon rabies: A time-space clustering approach. *Preventive Veterinary Medicine*, *56*(1), 89–103. doi: 10.1016/S0167-5877(02)00126-5

Urban, D. (1970). Raccoon Populations , Movement Patterns , and Predation on a Managed

Waterfowl Marsh. *Journal Wildlife Management*, *34*(2), 372–382.
